# Supplementary material for: Bdelloid rotifers deploy horizontally acquired biosynthetic genes against a fungal pathogen
Source: Nat Commun. 2024 Jul 18;15:5787. doi: 10.1038/s41467-024-49919-1 (PMC11258130; doi:10.1038/s41467-024-49919-1)
Supplement: Supplementary file 1 — Supplementary Information [file 41467_2024_49919_MOESM1_ESM.pdf]

Supplementary Information for:

Bdelloid rotifers deploy horizontally acquired biosynthetic genes against a pathogenic fungus

Reuben W. Nowell[1,2,3,4], Fernando Rodriguez[5], Bette J. Hecox-Lea[5], David B. Mark Welch[5] Irina R. Arkhipova[5], Timothy G. Barraclough[1,2], Christopher G. Wilson[1,2,*]

[1] Department of Biology, University of Oxford; 11a Mansfield Road, Oxford, OX1 3SZ, UK.
[2] Department of Life Sciences, Imperial College London; Silwood Park Campus, Buckhurst Road, Ascot, Berkshire SL5 7PY, UK.
[3] Institute of Ecology and Evolution, University of Edinburgh; Ashworth Laboratories, Charlotte Auerbach Road, Edinburgh, EH9 3FL.
[4] Biological & Environmental Sciences, University of Stirling, Stirling, Scotland, UK, FK9 4LA.
[5] Josephine Bay Paul Center for Comparative Molecular Biology and Evolution, Marine Biological Laboratory, Woods Hole, MA, USA.

* Corresponding author: chris.wilson@biology.ox.ac.uk

Contents:

Supplementary Methods 3
Supplementary Figures 15
Supplementary Tables..34
Supplementary References...50

Supplementary Methods

Rotifer and pathogen isolates

Animals belonging to the species *Adineta ricciae*[1] and *A. vaga*[2,3] were isolated respectively in 1998 from mud in Australia[1] and ca. 1984 from moss in Italy[4] and propagated clonally in continuous long-term culture across several laboratories[5–8]. We use the code 'AD001' for this lineage of *A. ricciae*, and 'AD008' for *A. vaga*. These cultures are available on request, and were selected because annotated genome assemblies were available for both species at the time the experiments were conducted, denoted 'Ar18' for *A. ricciae* and 'Av13' for *A. vaga*[7,9]. Cultures were maintained in 60mm plastic Petri dishes in sterilised distilled water, fed with *Escherichia coli* (OP50) and *Saccharomyces cerevisiae* (S288c) and subcultured approximately once per month. They were stored at 20°C in an illuminated incubator (LMS, Kent, UK) with a 12:12 hour light:dark cycle.

The fungal pathogen *Rotiferophthora globospora*[10] was found attacking co-occurring rotifers of the genus *Adineta* in soil in northern New York[11]. A pure culture on potato dextrose agar (PDA) was obtained in December 2008 using methods described elsewhere[12], and deposited in April 2009 with the USDA Agricultural Research Service Collection of Entomopathogenic Fungal Cultures (ARSEF) for long-term cryogenic storage under the accession code ARSEF 8995. Frozen mycelium was retrieved and revived from this collection in 2013, and since maintained in serial subculture on PDA at 20°C with a 12:12 hour light:dark cycle and with transfers every 4 months. This pathogen strain has no recent history of laboratory co-passage with either of the two rotifer hosts tested here and all three were isolated on different continents. However, *R. globospora* appears to be globally distributed—it was originally described from New Zealand[10] and has also been recorded in Japan (CGW, pers. obs.), in both cases attacking *Adineta*. Therefore, both host species are likely to encounter this pathogen in nature, especially given the high dispersal capacity and global distribution of bdelloid rotifers[13–16].

Infection by *R. globospora* is initiated when a host ingests infectious spores (conidia), which are spherical and approximately 3.5µm in diameter[10]. These lodge in the mouth or oesophagus, at which point the animal stops feeding and contracts within a few minutes (Fig. 1a, main text). Over the next 6–8 hours, the conidium produces a thin germ tube that penetrates the gut wall and swells into assimilative hyphae, which begin to invade and digest the surrounding host tissue after about 12–24 hours, as the infection becomes established. By 36–48 hours, fungal hyphae have filled most of the body cavity and the host is dead. Between 48–72 hours, hyphae begin to emerge through the host integument, and will eventually differentiate to form two types of spores: a handful of thick-walled resting spores, and hundreds of fresh infectious conidia (Fig. 1a, main text).

Because *Rotiferophthora* can only complete its life cycle by killing its host, these fungi are inherently highly virulent pathogens, and have been described as "devastating" to populations of *Adineta*[10]. *R. globospora* (ARSEF 8995) has been shown to exterminate laboratory populations of a sympatric *Adineta* clone within 28 days of initial exposure to a low density of conidia[11]. However, even if rotifer individuals are inevitably killed once the pathogen has established a large-scale infection within the body, an individual host can resist the initial attack and prevent an ingested spore from establishing a successful infection, or at least delay its progression. Even partial resistance at an early stage could dramatically slow the spread of an epidemic[17], giving clonal relatives time to escape the infested habitat[16,18] before the local population is exterminated.

Infection assays

To quantify resistance to *R. globospora* under standard conditions, rotifers were transferred by pipette from stock populations to a 2mL droplet of sterile distilled water, where eggs, corpses and food from the cultures were washed away. Adult individuals were transferred to 96-well plates (Thermo-Fisher), with approximately 11 animals per well (mean: 11.0, SD: 3.5) in 60µL of sterilised, distilled water. Rotifers were counted using a compound microscope (Nikon Eclipse E400), noting whether each animal was active (feeding or locomoting, Fig. 1a), contracted (with head withdrawn) or dead. Animals that died during the transfer (<1.5% of the total) were physically removed where possible, or recorded so they could be excluded from later counts.

To obtain pure suspensions of conidia, approximately 5x5mm of freshly subcultured sporulating mycelium on PDA was moved to 500µL of sterile distilled water in a 1.5mL Eppendorf tube. After vortexing, 400µL of conidial suspension was transferred to a fresh sterile tube and conidial density was measured using a haemocytometer and lactophenol cotton blue stain. An inactivated inoculum was prepared simultaneously as a control, by exposing an aliquot of conidia in water to a germicidal ultraviolet lamp (25W, 253.7nm) at a distance of 10cm for 45 minutes, with regular vortexing to ensure all spores were irradiated. This treatment was successful, as none of the rotifers in control wells treated with irradiated spores became infected.

Wells were inoculated with 8µL of freshly prepared conidial suspension at a density of 125 spores μL^{-1}. Negative control wells received 8µL of distilled water or inactivated spore suspension. The final density of spores in each well was high (ca. 15 conidia μL^{-1}) to ensure every animal was exposed to the pathogen in a synchronised pulse. This appeared to work, because 94% of animals in experimental wells were contracted 8 hours after exposure, versus a baseline of 3% of animals contracted in control wells (Supplementary Fig. 16). To check our inference that contracted animals have ingested fungal spores, small numbers of contracted rotifers (n =15) were isolated from wells between 4 and 7h after inoculation, transferred to microscope slides, squashed and imaged using phase contrast optics (as in Supplementary Fig. 1). All animals were found to have ingested spores: 12 animals (80%) had one spore; two animals (13%) had two spores, and one animal (7%) had ingested three spores. Wells from which animals had been removed for this purpose did not contribute to the experimental dataset.

Following inoculation, plates were stored in incubators (LMS 300NP) at 20°C, with a 12:12 hour light:dark cycle. After 8, 24 and 72 hours, rotifers were counted again, and classed as active, contracted, killed by infection or otherwise dead. A rotifer was considered to be killed by infection if at least one hypha had emerged through the integument from the interior. This criterion was unambiguous, in contrast with the difficulty of determining whether a fungal infection had established inside a contracted animal. By 72h, the proportion of infected animals in experimental wells appeared to have stabilised (Supplementary Fig. 16). In a subset of wells recounted at 96h, only a small fraction of animals (~6.5%) had newly developed visible infections. The relative risk of infection for *A. vaga* versus *A. ricciae* at 96h (RR 2.94, 95% CI 1.93–4.46; $Z = 5.06$, $P < 0.0001$) had not narrowed significantly since 72h (ratio of RR 0.78, 95% CI 0.47–1.31, $Z = –0.926$, $P = 0.354$), but the survivors had reproduced, making further tracking of the originally exposed cohort difficult. We therefore took the 72h timepoint as the standard measure of infection mortality. Individuals in negative control wells never became infected, whether they received water or sterilized spores, and the background death rate was ~2%.

Inoculation trials with both *A. ricciae* and *A. vaga* were replicated on multiple occasions using at least three separate source dishes for each species prepared at different times, and separately prepared cultures and inocula of fungi, with each set of trials replicated in at least three wells with accompanying negative controls in a blocked design. The total number of animals exposed across counted trials was 216 for *A. ricciae* and 189 for *A. vaga* (Fig. 1b). Rates of mortality from *R. globospora* infection were consistent across these trials, as was the difference in susceptibility between the species (Fig. 1b, main text). One set of well trials was run simultaneously and in close linkage with the scaled-up RNA-seq experiment described below, using rotifers from the same source populations, inoculated with the same live and irradiated pathogen suspensions. This enabled the timings and presumed final outcomes of infections in the RNA tubes to be inferred, even though those animals could not be directly counted and were sacrificed for RNA by 24h. The results of the RNA-linked well experiments were similar to earlier trials: mean infection mortality at 72h was 18% for *A. ricciae* and 79% for *A. vaga*.

RNA-seq experimental design

Rotifers for the RNA-seq experiment were reared at scale in eight replicate Petri dishes per species, with ca. 50 founders per dish, all from the same clonal laboratory line of each species. These were fed only with *E. coli* (OP50, 5×10^8 cells per dish) in distilled water. *S. cerevisiae* was omitted to avoid introducing further eukaryotic transcripts, and because gene expression by rotifers metabolising a fungal food source might complicate inferences about transcription in response to a fungal pathogen. The omission of *S. cerevisiae* as a food did not affect infection outcomes, because results of RNA-linked well assays were almost identical to earlier trials. When the *A. ricciae* dishes were initially established, each was inoculated with 50µL of 5µm-filtered water from the *A. vaga* source population, and vice-versa, to homogenise any co-cultured bacterial communities whose composition might differ between the cultured lines of the two species, and limit this as a potential source of gene expression differences. Dishes of the two species were stored in evenly interspersed blocks while the populations were growing.

Rotifers were counted and harvested after 4 weeks, when the mean population size was about 3000 per dish for *A. ricciae* and 2000 for *A. vaga*, which reproduces more slowly[19]. Each dish was gently washed, using several water changes to float and pour off eggs, bacterial cells and corpses, while active animals adhered to the plastic. Cleaned rotifers were detached from the plastic by dropping distilled water onto them from a 10mL syringe at 40cm height, followed by repeated aspiration and forcible expulsion of medium using a P1000 pipetter and a 1mL tip. Physical detachment was preferred over a salt or cold shock approach[7], to reduce the chance of inducing transcriptomic, immunological or behavioural changes. Suspended rotifers were poured into a 50mL centrifuge tube and pelleted at 3000 x g for 5 minutes using a swing-bucket cradle. All but 17.5mL of supernatant was removed, then a vortex shaker was used to resuspend the rotifer pellets and 1mL was transferred immediately by pipette to a 1.5mL Eppendorf tube. This process was repeated to yield 16 replicate 1.5mL tubes for each species. Each tube was centrifuged at 17000 x g for 3 minutes, and all but 100µL of water was removed from the rotifer pellet. The pelleted rotifers were left overnight to recover and redistribute themselves around the submerged interior of the tube. We estimate that the efficiency of rotifer recovery via this method is about 70%, based on numbers of animals left over in plates or tubes, giving approximately 1000 animals per tube for *A. ricciae* and 600 for *A. vaga*.

The 16 tubes for each species were randomly allocated to receive either live or irradiated pathogen spores, and to have RNA extracted either 7 or 24 hours later, with each

combination replicated four times. Irradiated spore suspensions, as described above, were used as a control treatment to account for physical, chemical, or nutritional effects of ingesting fungal cells, so that all else was equal except for pathogen viability. RNA sampling times were chosen to correspond to the early stages of infection[12]—by 7h, germ tubes from ingested spores have attempted to infiltrate the host, but assimilative hyphae would not yet be established. By 24h, assimilative hyphae would be present if the germ tube had been successful, but these would not yet have colonised the host extensively or killed it (Supplementary Fig. 1). Each tube was inoculated with 20µL of live or irradiated spore suspension at a density of 500 spores μL^{-1}, for a total of 10,000 spores and a final density of 80 conidia μL^{-1}, to ensure every animal was exposed as synchronously as possible. Tubes were incubated upright at 20°C in a blocked layout until RNA extraction.

To check the efficiency of spore exposure in tubes, a small subsample of rotifers from each tube was examined microscopically, starting 40 minutes after inoculation, and counted blind and in random order with respect to treatment, but blocked with respect to rotifer species. The *A. vaga* count was completed within 20 minutes and the *A. ricciae* count within 30 minutes. Proportions of contracted animals were then calculated, comparing the animals in treatment tubes to those that had received irradiated spore suspension. For *A. ricciae*, the proportion contracted in treatment tubes was 97.7%, and for *A. vaga*, this was 95.8%. This difference between species was not significant (χ^2, = 1.271, d.f. = 2, P =0.26, n =484). The UV-irradiation treatment appeared highly effective, with < 3% of contracted animals observed in control tubes for both species, consistent with results from the well trials described above.

RNA extraction and sequencing

Total RNA was extracted from each tube at the appropriate timepoint using a column-based RNeasy Mini kit (Qiagen #74104), following the manufacturer's protocol for animal tissues. To lyse and homogenise the rotifers, tubes were centrifuged at 17,000 x g for 3 minutes and 100µL of excess water was removed, leaving rotifers pelleted in approximately 20µL of water. After adding 50µL of Buffer RLT, the pellet was immediately disrupted and homogenised for 1 minute by pulsed application of a pellet pestle attached to a cordless motor (Kimble). A further 250µL of Buffer RLT was added to stabilise the lysate and rinse residue from the pestle, before proceeding with the manufacturer's protocol, including an on-column DNase I digestion step. Lysis and stabilisation were completed for all tubes within 30 minutes of the target timepoint, in a balanced order with respect to treatment and species.

RNA was eluted in 32µL of RNase-free water and 1.5µL aliquots were analysed using a Nanodrop 2000 (ThermoFisher). Spectrophotometric measurements were used to select the three replicates with the highest RNA concentrations from each treatment group for further analysis and sequencing, so that downstream steps had three biological replicates. These 24 tubes were frozen at -80°C and shipped on dry ice to the University of Edinburgh, where further quality control was undertaken by Edinburgh Genomics, including RNA quantitation with a Qubit 2.0 fluorometer in duplicate for each sample, and RNA ScreenTape analysis with an Agilent 2200 TapeStation system to assess RNA integrity. All 24 samples proceeded to cDNA library preparation, using the TruSeq stranded mRNA kit (Illumina) to enrich for polyadenylated transcripts. The indexed ~200 bp insert libraries were sequenced in multiplex on an Illumina NovaSeq 6000 at Edinburgh Genomics, using an SP flow cell to generate 50-base paired-end reads.

Data filtering and quality control

The 24 libraries yielded 102.9 Gb of raw sequencing data. Raw sequencing reads were quality- and adapter-trimmed using BBTools 'bbduk' v38.73 (parameters 'ktrim=r k=23 mink=11 hdist=1 tpe tbo'), and error-corrected using BBTools 'tadpole' (parameters 'mode=correct tossjunk tossuncorrectable') (https://sourceforge.net/projects/bbmap/). Unwanted reads derived from ribosomal RNA (rRNA) were removed by mapping the data to the SILVA rRNA database[20] using BBTools 'bbmap' with parameters 'local=t outu=filtered_R#.fq.gz' (i.e. retaining only unmapped pairs of reads). Sequences derived from the fungal pathogen were removed using the same approach, mapping to sequenced genomes of fungi in the family *Clavicipitaceae* (NCBI taxid 34397). The proportion of remaining reads mapping to the Ar18 or Av13 target genomes was assessed using STAR v2.7.3a[21] with the parameter '--twoPassMode Basic'. Final data quality was assessed visually using FastQC v0.11.6[22] and MultiQC v1.9[23]. All raw sequencing data have been deposited in the relevant International Nucleotide Sequence Database Collaboration (INSDC) database with the Study ID PRJEB39927 (see Supplementary Table 9 for run accessions).

Before using the data to quantify expression of horizontally acquired genes encoded by rotifers, it was important to account for reads from contaminating microorganisms in the non-axenic experimental tubes. For example, bacterial contamination might complicate expression measurements for rotifer HGT_C genes that share partial homology with true bacterial sequences. Rotifer-encoded HGT_C genes also preclude simply filtering out all reads mapping to known bacterial sequences. These potential issues were instead assessed by focusing on the *E. coli* that was fed to the rotifers (strain OP50), whose genome sequence is available (GenBank accession GCF_009496595.1). Despite efforts to wash the animals and allow time to clear the gut, OP50 was still by far the largest potential source of contaminating transcripts—approximately 3.56% (± 2.8 SD) of unfiltered reads mapped to the OP50 genome (Supplementary Table 11). In filtered reads, however, this was reduced to 0.016% (± 0.015 SD; Supplementary Table 12), indicating that the vast majority of sequenced OP50 reads were derived from 16S and 23S rRNA, as confirmed by further direct mapping to OP50 rRNA (Supplementary Table 13). *E. coli* therefore made a negligible contribution to the mRNA pool, probably owing to massive enrichment for eukaryotic sequences via the poly-A selection step. Nevertheless, to exclude even a marginal effect of mis-mapped bacterial reads on estimated HGT_C expression, each significantly upregulated HGT_C gene was examined for any contribution from OP50-derived reads. For none of these genes did more than one or two reads from OP50 contribute to expression calculations, whereas the corresponding mean number of total reads was 13655 (minimum 153) (Supplementary Tables 14 and 15). Given that *E. coli* OP50 was the most common contaminant, its negligible contributions to mRNA-derived reads and HGT_C expression calculations indicate that the data and results reflect genes encoded and expressed by rotifers, and not RNA originating in bacterial contaminants. The hypothesis that HGT_C sequences arise from contamination is further refuted by the presence of corresponding paired copies on genomic scaffolds for *A. vaga* and *A. ricciae* whose divergence matches the rest of the genomes, by the fact that investigated HGT_C genes often show a high degree of divergence from the nearest sequenced non-metazoan copies, and by the presence of introns in genes encoding proteins whose closest homology is otherwise to bacteria. Many upregulated gene copies were closely related to each other and showed patterns of homologous or homoeologous relationships consistent with the ancestral tetraploidy of bdelloid species[24,25].

After data filtering and quality-control, 78.5 million reads were retained per library (94.2 Gb total data, Supplementary Table 9). Over 99% of filtered reads mapped to the Av13 and Ar18 reference genomes. To check for overall agreement between the RNA-seq data and

transcriptomes predicted from whole genomes, the filtered data were *de novo* assembled using the Trinity software v2.8.9[26,27], resulting in 118,860 and 63,749 transcripts representing 41,527 and 35,079 'genes' for *A. ricciae* and *A. vaga*, respectively (Supplementary Table 10). Transcriptomes showed G + C proportions that were consistent with their respective genomes (*A. ricciae* = 37.6%; *A. vaga* = 33.0%) and high 'completeness' scores as measured by BUSCO analysis (>97% of core eukaryote genes completely recovered in both cases). Transcript-to-genome mapping rates were >99% in both species, with 87.1% (*A. ricciae*) and 81.8% (*A. vaga*) of introns correctly called based on previously published gene models. Note that *de novo* transcriptomes were not used for subsequent gene expression analyses, which used gene models predicted from whole genomes instead (see the following section).

Differential expression analysis

Transcript quantification was performed using Salmon 'quant' v0.14.1[28], with the gene models of Nowell et al. (2018)[9] as the target transcriptomes. Short transcripts <150 bp were removed before analysis, resulting in a total of 58,423 (*A. ricciae*) and 66,273 (*A. vaga*) gene models to test for differential expression. Genomic scaffolds were appended as 'decoys' to each transcriptome prior to quantification as recommended in the Salmon documentation. Relationships between biological replicates within and between samples were visually checked using utility scripts in the Trinity software[26,27], with PCA results indicating high correlation in gene expression among replicates (Supplementary Fig. 17). Statistical analysis of the resulting count matrix was performed with DESeq2 v1.26.0[29], which uses negative binomial generalized linear models to test for differential expression. *P*-values were adjusted for multiple testing using the Benjamini-Hochberg method[30] to control the false discovery rate (FDR). Stringent thresholds of FDR < 1e–3 and $\log_2$ fold-change > 2 (i.e. 4-fold expression difference) were used to define differentially expressed genes for downstream analysis. Comparison of control populations (Supplementary Figs. 18 and 19) showed that HGT_C genes are more likely to be expressed than ancestrally metazoan genes under control conditions, but at significantly lower levels. The enrichment we saw for HGT_C among differentially expressed genes is thus unlikely to be due to a known false-positive bias in some RNA-seq analyses toward genes with higher expression levels[31,32]. However, to test for this effect and check for overall consistency in our results, we also calculated differential expression using two other software packages: edgeR v3.28.1[33–35] and limma/voom v3.42.2[36]. These handle known biases in RNA-seq analysis differently[32], enabling us to establish that the key differential expression result does not depend on the software used (Supplementary Figs. 7–10).

Gene sharing among the different treatment groups was visualised using the R packages 'ggvenn' v0.1.10 and 'eulerr' v7.0.0, linked by orthogroup (see below) when compared across species.

Gene orthology

Orthologous relationships between *A. ricciae* and *A. vaga* genes were determined using OrthoFinder v2.3.12[39] with default parameters. Proteomes from 33 other rotifer species[9,40,41] (i.e., all available transcriptome data) were included in the analysis to aid orthology inference (see Supplementary Data 8 for full description of included genomes). Clustering produced a total of 15,585 orthologous groups (OGs) that contained at least one gene from both species, accounting for 86.5 and 81.7% of total genes in *A. ricciae* and *A. vaga*, respectively. Resulting orthogroups were used to link genes across *A. ricciae* and *A. vaga* for assessment of correlations in gene expression between the two species (e.g., PCA below and Supplementary Figs. 3 and 4).

Principal component analyses

To compare gene expression dynamics across species and experiments, a mean expression level was calculated for each orthogroup in each replicate in each species, by taking the mean value of normalised counts per million (CPM) gene expression data (from the DESeq2 results, as used for cross-replicate PCA by Trinity QC scripts) across all genes within that orthogroup as defined above (Supplementary Data 2). PCA was then performed using the 'prcomp' function in R, with parameters 'center=F scale.=F'. To compare the relative magnitude and consistency of gene expression changes occurring within the pathogen and desiccation experiments, we plotted replicates from the two datasets on the same set of principal component axes (Supplementary Fig. 11).

HGT classification

Putative HGT candidate genes (denoted HGT_C) were determined from the recent analysis of Jaron et al.[42]. Briefly, proteins were aligned to the UniProt90 sequence database[43] using Diamond 'blastp' v0.9.21[44] with the parameters '--sensitive -k 500 -e 1e-10'. For each protein, a HGT 'index' (h_U) was computed based on the alignment bitscores to the best-matching hits from the Metazoa (B_{IN}) and non-Metazoa (B_{OUT}) with the formula: $h_U = B_{OUT} - B_{IN}$[45]. A 'consensus hit support' (CHS) score was also calculated as the proportion of secondary hits in agreement with the result based on the h_U[9,46]. An initial set of HGT_C were defined if $h_U > 30$ and CHS > 90%, but removing a previous filtering step of requiring co-assembly of the HGT_C on a contig which also encodes a gene of 'unambiguous' metazoan origin. Initial investigations found this criterion to be overly conservative: *bona fide* HGT_C were being excluded because they were encoded on short scaffolds with no other genes, thus failing the filter. Instead, we filtered this initial set based on tBLASTn alignment to a recently published, near-chromosomal level *A. vaga* assembly[47] ('Av20', diploid version), retaining only those with a good match (E-value ≤ 1e–5) to this genome. This allowed us to retain HGT_C clearly encoded on chromosomal scaffolds for subsequent analysis while removing potential contaminants from the Av13 gene model set. Phylogenetic trees of selected HGT_C and their putative co-orthologs from the UniRef90 database were constructed using IQ-TREE v1.6.12 with the parameters '-alrt 1000 -bb 5000 -m TEST'[48–50].

HGT_C presence or absence in other bdelloid species

The presence or absence of significantly upregulated HGT_C across bdelloid genera was tested explicitly by searching (using Diamond 'blastp' as above) for significant hits to the combined proteomes of all bdelloid samples with available whole-genome data. The number of hits with minimum identity = 40% to other proteomes are tabulated and summarised in Supplementary Data 12.

Functional annotation

Protein sequences from the *A. ricciae* and *A. vaga* reference genomes were aligned to the SwissProt[43] and Pfam[51] sequence databases using BLAST v2.10.1+[52] and HMMER v3.3 (http://hmmer.org/) respectively. Signal peptide cleavage sites and transmembrane helices were identified using SignalP v4.1[53] and TMHMM v2.0[54] respectively. Protein domains were further identified using InterProScan v5[55]. Functional annotations were assimilated using the Trinotate v3.2.0[56] pipeline.

Functional enrichment analyses

Functional enrichment analysis was performed using GOseq[57], based on gene ontology (GO) terms identified during functional annotation above. For each timepoint, the test set was defined as HGT_C that were significantly up- or downregulated (based on absolute fold-change > 4 and FDR < 1e–3) versus the background set of the whole genome. GOseq was run using the ‘run_GOseq.pl’ utility script in the Trinity software package. At the most stringent threshold (FDR < 0.001), 29 GO terms were significantly enriched for genes upregulated by *A. ricciae* at T24 (rising to 43 and 68 for FDR < 0.01 and < 0.05 respectively). Significant GO terms were visualised using Revigo[58] (default parameters). To assist in interpreting enriched GO terms, each unique HGT_C associated with significantly enriched terms was individually translated and processed as a BLASTp query against the UniProt database, with rotifers (NCBI taxid 10190) excluded. For clarification in selected cases, a tBLASTn query was run against the NCBI nucleotide database. The list of significantly enriched GO terms that appeared to point to NRP/PKS functions was: GO:0031177 (MF) phosphopantetheine binding; GO:0017000 (BP) antibiotic biosynthetic process; GO:0016999 (BP) antibiotic metabolic process; GO:0072341(MF) modified amino acid binding; GO:0017144 (BP) drug metabolic process; GO:0019842 (MF) vitamin binding; GO:0033218 (MF) amide binding; GO:0016853 (MF) isomerase activity.

We tested whether the apparent pattern of enrichment for NRP/PKS-related terms in *A. ricciae* versus *A. vaga* was affected by the reference genomes used for HGT_C and functional enrichment analyses, which were assembled and annotated using different methods for the two species. We repeated the functional enrichment analysis as above, using transcriptomes assembled and annotated de novo from RNA-seq data, independent of reference assemblies or HGT_C filtering (Supplementary Data 4). ‘Phosphopantetheine binding’ (GO:0031177) was still the most highly enriched term for *A. ricciae* (FDR = 3.72e–15), with ‘isomerase activity’ and ‘antibiotic biosynthetic process’ also significantly enriched (FDR = 0.0001), whereas none of these NRP/PKS-associated terms was enriched at any level for *A. vaga*. The same conclusion is therefore reached whether transcriptomes are assembled and annotated de novo from RNA-seq data, or mapped to the predicted proteome of existing reference genomes.

To account for the lower RNA-seq coverage and power to detect functional enrichment in the desiccation dataset[59], we relaxed the threshold for GO term enrichment to FDR < 0.05 (Fig. 4, main text), then to FDR < 0.1 (Supplementary Data 5). This ensured we would not miss weak signals of functional overlap between HGT_C genes upregulated in response to biotic and abiotic stress. Even at this lowest threshold, however, only five of the 54 significantly (FDR < 0.05) enriched GO terms for pathogen-exposed *A. vaga* at T24 (Supplementary Data 3) overlapped with desiccation stress, all unrelated to the putative defensive functions discussed elsewhere: three redundant GO terms for oxidoreductase activity, ‘trimethyllysine dioxygenase activity’, ‘iron ion binding’ and ‘carnitine biosynthetic process’ (which is not mediated by NRP/PKS). Likewise, no enrichment for NRP/PKS-related GO terms and no upregulation of putative NRP/PKS CDS was reported in a separate transcriptomic analysis of desiccation and radiation stress in *A. vaga*[60].

The list of significantly enriched terms pointing to RNA ligase functions was: GO:0003972 (MF) RNA ligase (ATP) activity; GO:0008452 (MF) RNA ligase activity; GO:0042245 (BP) RNA repair; GO:0098004 (BP) virus tail fiber assembly; GO:0016886 (MF) ligase activity, forming phosphoric ester bonds. None of these terms was significantly enriched in our analysis of the response to desiccation, nor were they enriched (FDR < 0.05) in a separate analysis of desiccation and ionizing radiation in *A. vaga*[60], but two individual RNA ligases of horizontal origin were reported as upregulated in response to radiation.

Screen for putative NRP/PKS and genomic validation in *A. vaga*

An automated screen was conducted on the predicted proteomes of *A. vaga* and *A. ricciae* for putative NRP/PKS genes using a conservative approach based on the presence of canonical adenylation (AMP-binding, Pfam accession PF00501), thiolation and peptide carrier protein (PP-binding, PF00550) and condensation (PF00668) domains, using HMMER 'hmmsearch' v3.3 (http://hmmer.org/). Only proteins with significant matches (E-value ≤ 1e–5) to all three domains were classified as putative NRP/PKS in this 'three-domain' set of gene models. The same survey was conducted on published proteomes of the monogonont rotifers *Brachionus plicatilis* (GenBank: GCA_003710015.1) and *B. calyciflorus* (GCA_905250105.1), and the acanthocephalan rotifer *Pomphorhynchus laevis* (GCA_012934845.2); none were found. To check this set against the genomic repertoire of NRP/PKS clusters, a more inclusive manual survey was conducted in *A. vaga*, where a chromosome-scale haploid genome assembly (Av20) is available[47] alongside the diploid reference assembly used elsewhere (Av13). First, Av13 was screened for putative biosynthetic clusters based on the presence of adenylation (A-) and condensation (C-) domains using tBLASTn, agnostic to prior annotations. The number of individual domains occurring once per module was counted, to obtain an independent estimate of the number of modules while accounting for partially assembled NRPS genes that may be distributed between scaffolds. This yielded a total of 306 hits to A-domains and 276 hits to similarly sized C-domains. Since NRPS are organized as iterative modules, each scaffold represented by a set of A-domains and/or C-domains was manually curated to obtain the putative multi-domain cluster. A total of 110 gene models corresponding to putative biosynthetic clusters were identified, most of which exhibit higher similarity to bacterial than to fungal NRPS, alongside 12 annotated polyketide synthases (PKS), with some of the annotated clusters representing NRPS-PKS hybrids. This list recovers all members of the conservative three-domain set (n = 60), but also many fragmented or potentially non-biosynthetic annotations, lacking one or more canonical domains.

Cross-referencing to Av20 was performed using a multi-step approach. First, the software AntiSMASH v6.1.1[61] was used for automated detection; this revealed potential NRPS-PKS cluster locations, but gene annotations were not optimal, especially intron detection, since the method is optimized for fungal or bacterial genomes. Further searches used tBLASTn with translated CDS of the 122 NRP/PKS-related annotations from the maximal Av13 set. Additionally, putative NRPS A-domains were confirmed with NRPSpredictor2[62], which uses support vector machines to predict substrate specificity based on the configuration of the amino acid residues in the active site of an A-domain, with Av20 haploid gene annotations showing a total of 51 with substrate predictions consistent with A-domains. In combination, these lines of evidence identified approximately 40 putative biosynthetic gene clusters in the haploid Av20 assembly (Supplementary Data 9), distributed across the six chromosomes (Fig. 5c, main text). Approximately 10 appeared defective in some way (3' incomplete; apparent frameshifts in key modules), but several of these CDS were highly expressed, so these apparent defects may reflect ongoing assembly and annotation challenges even in a high-quality genome. Clusters (especially those consisting of more than one module) are preferentially located in subtelomeric regions, which agrees with the known enrichment of HGT_C in telomeric and subtelomeric regions in the *A. vaga* genome.

All of the Av13 annotations in the three-domain NRP/PKS set (n = 60) could be matched by BLAST to one of 20 clusters in Av20. Each matched cluster attracted a median of 2 annotations, as expected when mapping to a haploid assembly, but with some variability. For example, one especially large subtelomeric Av20 cluster (3.2; 108.5 kb) had 8 matching Av13 annotations, perhaps reflecting scaffold fragmentation in the Av13 assembly, while two focal annotations (AVAG|g23567 and AVAG|g48151) mapped uniquely to a single Av20

cluster each (3.4 and 5.2 respectively), suggesting unresolved alleles in Av13. The remaining Av20 clusters (*n* = 19) did not attract matching annotations from the three-domain set, largely because they lacked the canonical C-domains required in the automated survey. Of the 18 Av20 clusters with annotated C-domains, 17 (94%) were captured in the three-domain set.

The genomic context of identified clusters was ascertained directly for *A. vaga* using the Av20 assembly. Of 40 putative NRP/PKS clusters shown in Fig. 5c, 27 (67.5%) are within a subtelomeric region, as defined by a mean relative density of >10 telomeric repeats assessed in a 1 Mb sliding window (Supplementary Fig. 12; see also Figure S10 of Simion et al. 2021). For both species, a statistical approach was used to confirm subtelomeric location, by counting the frequency of three telomere-associated genome features (the telomeric repeat hexamer "TGTGGG"[63], low gene density and elevated transposable element repeat density) in a (maximum) 50 kb window surrounding each putative NRP/PKS gene model, using BEDTools v2.29.2. The span of each feature was converted to a proportion by dividing by the actual window size for each flanking region, to correct for window size variation. The genomic context of eukaryote BUSCO genes (*n* = 303) was evaluated for comparison. Relative to BUSCO genes, NRP/PKS CDS in both rotifer species show significant associations with increased telomeric repeats (estimate = 0.72, SE = 0.05, $P < 0.001$) and transposable element repeats (estimate = 1.33, SE = 0.11, $P < 0.001$), and decreased gene density (estimate = –0.09, SE = 0.01, $P < 0.001$; Supplementary Fig. 13 and Supplementary Table 6).

One focal NRP/PKS annotation (AVAG|g23567) was not located in a subtelomeric region of Av20, but approximately 6.8 Mb along Chromosome 3 (Fig. 5c; cluster 3.4). It was also unusual among upregulated annotations in apparently lacking orthologous copies in other bdelloids (Supplementary Data 12). Contamination can be excluded, as it was assembled almost identically in Av13 and the Hi-C-scaffolded Av20, with consistent linkage to flanking metazoan genes. The cluster also has few or no introns. Together, this evidence suggests a recent acquisition. We further tested this hypothesis by measuring the guanosine-cytosine (GC) content of the cluster relative to the local genomic context. Elevated GC content is predicted for recently acquired sequences[7] that have yet to converge with the genomic background. We tested this for AVAG|g23567 by calculating %GC in a 20kb sliding window along its Av13 contig (AVAG00146). The focal annotation sits at the global maximum of the resulting landscape (Supplementary Fig. 15); no equivalent window in the 365 kb contig shows such high GC content (38%). We used the same method to assess %GC in Av20, where AVAG|g23567 corresponds to cluster 3.4 on Chromosome 3 (the longest chromosome, 20.35 Mb). Again, the maximum GC content (38.1%) for this contig occurs within the focal NRP/PKS cluster. The next-highest 20kb window has a substantially lower value (37.2%). Cluster 3.4 thus underpins the most marked peak in GC content seen on Chromosome 3, consistent with other evidence that it has recently been acquired from a bacterial genome.

Phylogenetic analyses

Phylogenetic trees of rotifer-encoded NRP/PKS proteins with bacterial and fungal homologs were constructed based on the alignment of the condensation domain (PF00668) with Pfam 'seed' representatives and selected best-matching (i.e. lowest *E*-value) homologs from UniProt/Uniref90 (Supplementary Data 8). Note that the sequence for ARIC|g51138 shown in Fig. 5b was not aligned as it lacks the focal C domain, which is encoded instead in the adjacent gene model (ARIC|g51137). Searches were conducted using Diamond 'blastp' v2.1.8[64] with the parameters "-k 50 --taxon-k 1 --taxon-exclude 44578 -e 1e-5", i.e. excluding any hits to sequences from the Class Bdelloidea but allowing hits to other metazoans should they exist. Additional sequences from other taxa were added manually for some analyses, e.g., rare cases of NRP/PKS in animals[65] to Fig. 5a. Alignments were built using HMMER

‘hmmalign’, and phylogenetic analysis performed using IQ-TREE v1.6.12[48], using ModelFinder for automatic model selection[49] and the ultrafast bootstrap[66] and SH-like approximate likelihood ratio test[67] for inferring branch support (parameters ‘-m TEST -bb 2000 -alrt 1000’). Resultant trees were plotted in R using the ‘phytools’ v0.7-70[68] and ‘ape’ v5.4.1[69] packages. Aligned rotifer proteins generally show low similarity to even the best-matching bacterial hits (e.g., pairwise BLSM62 residue matches are < 50%, even where long sequences spanning multiple domains can be aligned).

Orthology and copy number estimate for the upregulated NRP/PKS cluster

To test for orthology between AVAG|g48151 and the upregulated, putatively fragmented *A. ricciae* CDS (Fig. 5a and b, main text), the corresponding regions were examined in Av20, and in an alternative short-read genome assembly for *A. ricciae* (denoted Ar21[41]). Although internal sections of the putative NRPS-PKS hybrid cluster may have been collapsed where modules are identical, or fragmented into separately-assembled contigs where alleles diverge, the number of unique flanking sequences at the 5’- and 3’-termini of the gene model can be used to estimate the copy number for each cluster, even if the assembly is less reliable in the middle. We therefore inspected the adjacent flanking regions in the *A. vaga* and *A. ricciae* genomic contigs encoding the N-terminal and C-terminal regions of upregulated clusters, looking for local microsynteny in the 5’- and 3’-flanks. The single-copy AVAG|g23567 (scaffold AVAG00146 in Av13; Chr5: 665120..721107 in haploid Av20) does not have a recognizable ortholog in *A. ricciae* assemblies. The single-copy AVAG|g48151 in *A. vaga* (scaffold AVAG00591in Av13; Chr5: 665120..721107 in haploid Av20) has one true ortholog in *A. ricciae*: ARIC|g35898, as judged by the presence of a cytochrome P450 CDS in its immediate 3’-flank on the corresponding Ar21 genomic contig ARIC00373, and an ABC transporter B1-like CDS at the 5’-flank. It also has at least two potentially intact paralogs, indicated by upregulated CDS with alternative 5’ and 3’ flanks encoding different products: ARIC|g15363, with amiloride-sensitive sodium channel at the 5’-flank in contig ARIC00114; ARIC|g49019 with RNA-recognition motif RRM1_CPEB2-like protein at the 3’-flank in the Ar21 assembly contig ARIC003_00110, and ARIC|g51257, with ABC transporter B1-like protein at the 3’-flank in the Ar21 assembly contig ARIC003_01015; and AVAG|g51868 with MFS transporter-like abhydrolase domain-containing protein at the 5’-flank in the Ar21 assembly contig ARIC003_02601. An additional distinct 3’-flank was detected on a contig harboring an NRPS C-terminal fragment, however it was 5’-truncated and the copy was deemed non-functional. The remaining CDS from Fig. 5b belonging to the middle modules from the same cluster could not be confidently matched with contigs harboring flanking genes, and can be assumed to originate from collapsed or improperly assembled middle modules from one of the paralogs. Note that some copies may have undergone further tandem duplications, thus the current estimate of 3 (or 6, if both alleles are counted) represents the minimal number, which may be revised upwards when a chromosome-quality assembly becomes available. The difficulty in assembling and resolving so many divergent copies of a multimodular cluster (and potential accessory transporter genes) helps explain the apparent fragmentation into multiple gene models for *A. ricciae* (Fig. 5b). Nevertheless, even where multiple gene models overlap (e.g. ARIC|g35898; AVAG|g51868), each feature was assessed as being strongly upregulated in its own right, in analyses where all 11 CDS were available to attract RNA reads (Fig. 5d). Therefore, automated and manually curated annotations across multiple alternative genome assemblies support the conclusion that *A. ricciae* both encodes more copies of the focal NRPS-PKS cluster than *A. vaga* and upregulates these copies more strongly.

Secondary metabolite prediction

Secondary metabolite products of focal NRP/PKS gene models were predicted using the SeMPI v2.0 web server[70], with maximum cluster distance set to 25 kb and all metabolite databases selected, but otherwise default settings. We used the upregulated *A. vaga* annotation AVAG|g48151, because this corresponds to a full-length PKS-NRPS cluster whereas the orthologous annotations in *A. ricciae* were fragmented as discussed above. Two different sequences were submitted: an extraction of the region spanning the predicted gene model for AVAG|g48151 (23.4 kb including introns), and the full Av13 genomic scaffold on which this gene model is encoded (AVAG00591, 98.7 kb). The automatic reannotation performed as part of the SeMPI pipeline gave slightly different module and product predictions (gene model region: $C_{70}H_{86}N_8O_{26}$; full scaffold: $C_{64}H_{77}N_7O_{23}$), though both predictions had highest similarity to tyrocidines. Fig. 6a (main text) reports the prediction for the full scaffold input because this is agnostic with respect to prior annotations, but both outputs are provided in Supplementary Data 11 (interpretation of files and terms in this output can be found in the documentation supplied at: https://sempi-2-docu.readthedocs.io/en/latest/). For AVAG|g23567, the full scaffold (AVAG00146, 365.1 kb) was used. The predicted metabolite for AVAG|g48151 was initially rendered by SeMPI with chemical structural errors (pentavalent carbon atoms), so was re-exported in SMILES format to ChemDraw JS (version 19.0.0-CDJS-19.0.x.9+da9bec968, PerkinElmer Informatics), cyclicised with appropriate valences and re-rendered using RDKit.js (https://github.com/rdkit/rdkit).

Supplementary Figures

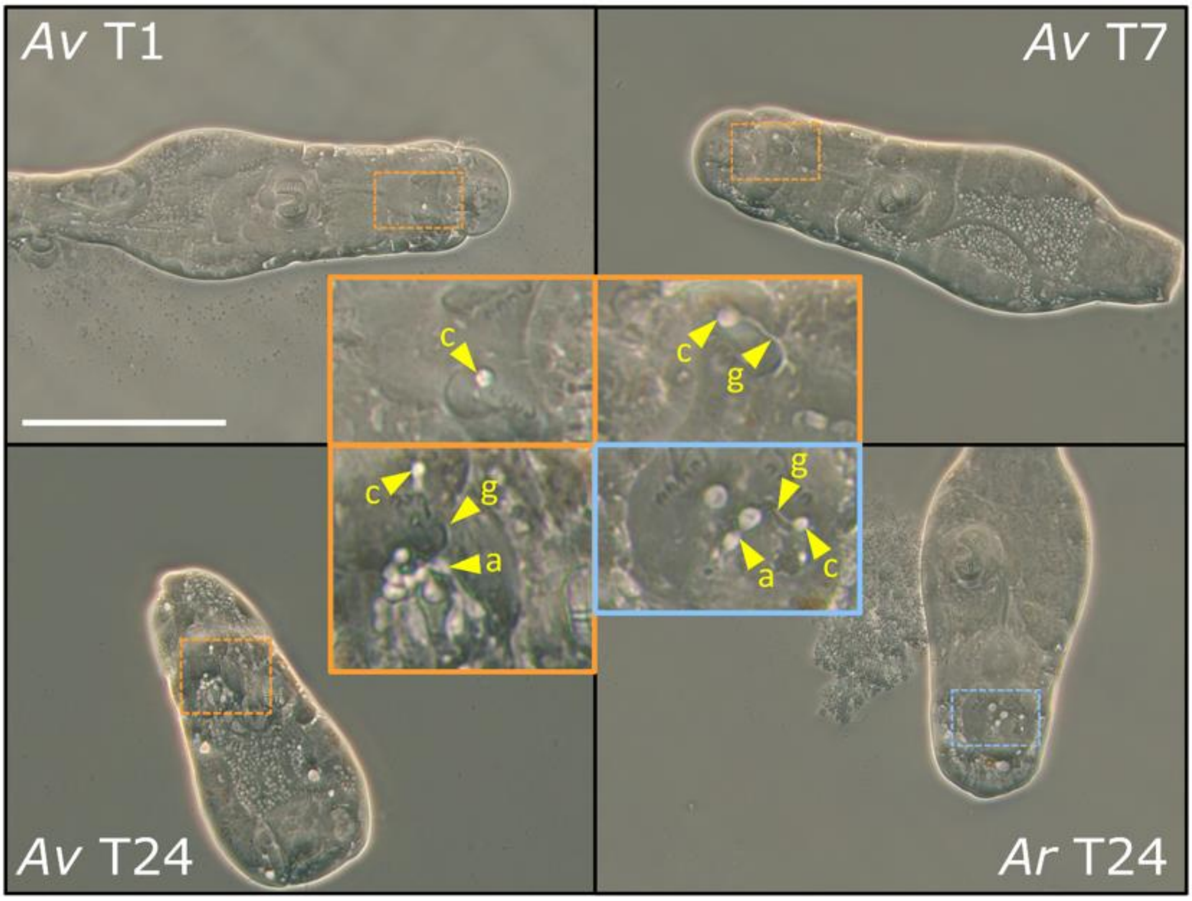

Supplementary Fig. 1. **Photomicrographs of four individual bdelloid rotifers, illustrating the typical visible timecourse of early attack by *R. globospora*.** Taken using a SPOT Insight 2MP Color Camera mounted on a Leica DM1000 compound microscope using phase contrast optics and a 40x objective, with pressure applied to partially squash the animal between a glass slide and a coverslip. Scale bar for outer panels is 100µm; inner panels show areas of detail (3x enlarged). ***Av* T1**: *Adineta vaga* 1 hour after exposure to spores, showing a conidium (c) lodged in the mouth near one of the bilateral rows of 4 'U-hooks' that form the rakes. Micrograph is representative of *n* = 9 replicate animals. ***Av* T7**: A different *A. vaga* 7 hours later, showing germination of a lodged conidium into a filiform germ tube (g) that has penetrated the oesophageal tissue and begun to bifurcate (*n* = 9 replicates). ***Av* T24**: After 24 hours, a germ tube from a conidium lodged near the rakes has penetrated the tissue between the mouth and trophi and has differentiated to form lobed, branching assimilative hyphae (a). Micrograph is representative of *n* = 7 replicate infections. ***Ar* T24**: An individual *A. ricciae* that was exposed to the same spore inoculum at the same time as *A. vaga*. After 24 hours, a similarly lodged and differentiated conidium can be seen, except that the assimilative hyphae in this case appear less extensive and have not penetrated as deeply beyond the mouth. Micrograph is representative of *n* = 5 replicate animals. It is unknown whether the animals seen here would have gone on to resist or succumb to the infections, because the slide squash is irreversible, but elsewhere we show that survival rates for *A. ricciae* are three times higher than for *A. vaga*.

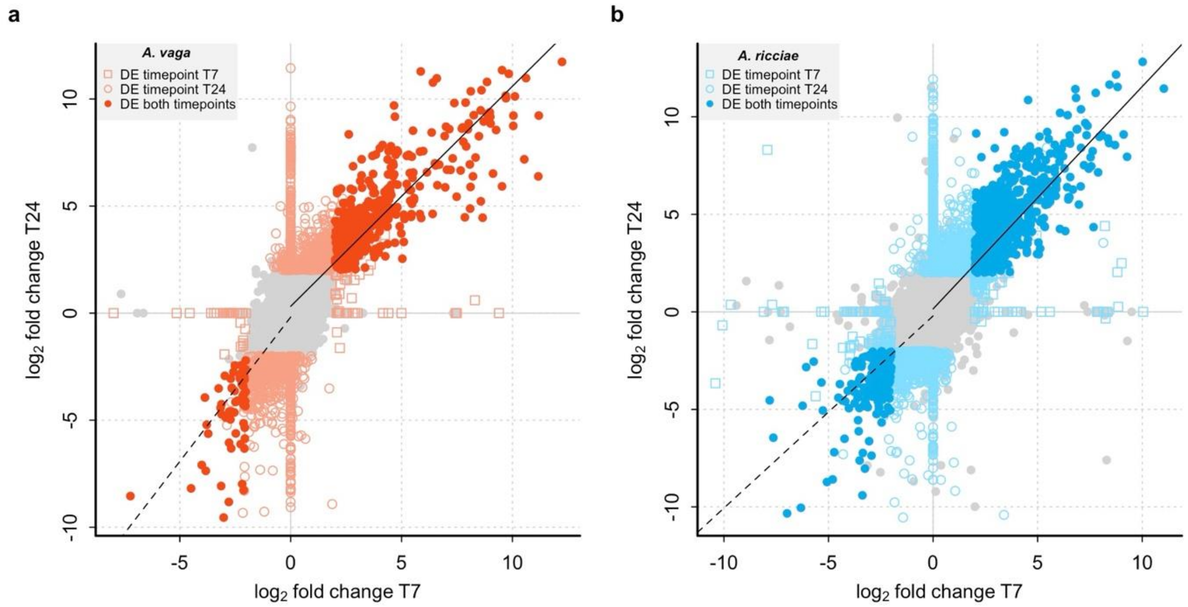

Supplementary Fig. 2. Correlation in DE between timepoints. Each point represents a single gene, and its $\log_2$ fold change in expression in treatment groups versus control groups at timepoint T7 (*X*-axis) versus timepoint T24 (*Y*-axis) for **a** *A. vaga* and **b** *A. ricciae*. Positive values represent upregulation in the treatment groups relative to control groups; negative values represent downregulation. Genes with significant DE in both timepoints are shown in red (*A. vaga*) and blue (*A. ricciae*); genes significant in one timepoint but not the other are shown with diamond and circle symbols (see legends). Genes with non-significant DE in both timepoints are plotted in grey. Solid black lines show the linear relationship for all upregulated genes (i.e., genes with $\log_2$ fold change > 0 in both timepoints; Pearson's correlation $R = 0.84$ and 0.82 for *A. vaga* and *A. ricciae*, respectively; $P < 2e{-}16$ in both cases). Dashed black lines show the linear relationship for downregulated genes ($\log_2$ fold change < 0 in both timepoints; Pearson's correlation $R = 0.67$ and 0.66 for *A. vaga* and *A. ricciae*, respectively; $P < 2e{-}16$ in both cases).

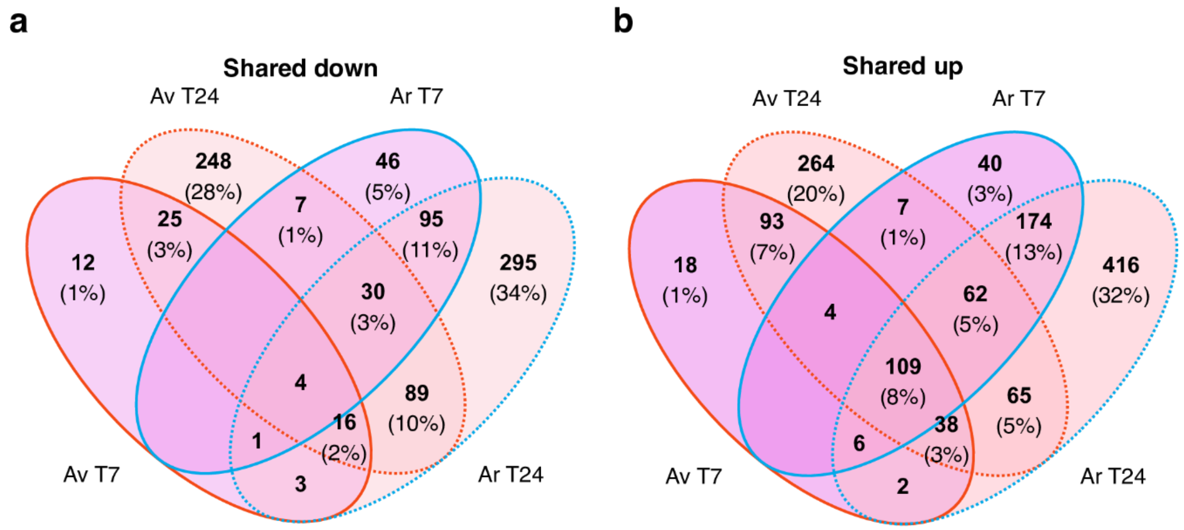

Supplementary Fig. 3. Extent of gene sharing in differentially expressed subsets across timepoints and species. Values in each segment show the number of genes significantly **a** upregulated and **b** downregulated for intersecting groups. Genes are linked by orthologous group ID (note that values represent numbers of orthologous groups, which do not always match the number of individual genes; see Supplementary Methods for more details). *A. vaga* and *A. ricciae* are shown in red and blue outlines, respectively; T7 is shown with a solid outline, T24 with a dashed outline. Segments with no values have no genes (orthogroups) shared across that intersection. No genes were shared between upregulated and downregulated sets for different timepoints for either species, so these two plots do not themselves overlap.

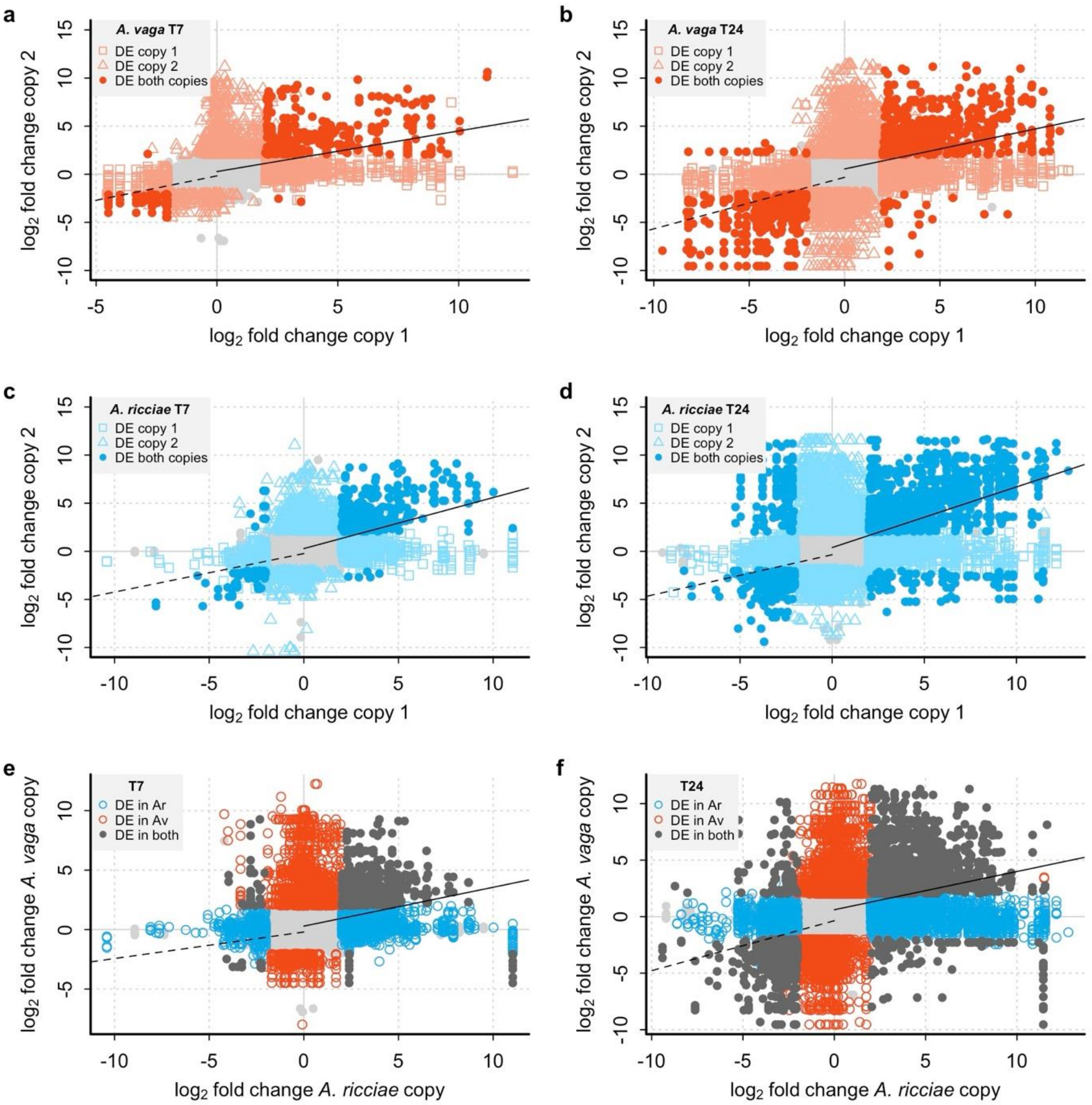

Supplementary Fig. 4. Correlation in DE between gene copies within and between genomes. Plots show the $\log_2$ fold change in expression in treatment versus control groups for **a** gene copies within *A. vaga* at timepoint T7, **b** gene copies within *A. vaga* at timepoint T24, **c** gene copies within *A. ricciae* at T7, **d** gene copies within *A. ricciae* at T24, **e** gene copies between species at T7, and finally **f** gene copies between species at T24. Note that each point represents a relationship between a pair of genes, not a gene itself (i.e., putative homologs, homoeologs or paralogs for within-genome comparisons, or orthologs for between-genome comparisons). Solid black lines show the linear relationship for all upregulated genes (i.e., genes with $\log_2$ fold change > 0 in both copies; Pearson's correlation R = 0.43, 0.43, 0.49, 0.60, 0.35 and 0.37 for *A. vaga* and *A. ricciae* within-genome copies (i.e., 'homologs'), timepoints T7 and T24 between-genome copies (i.e., 'homoeologs'), respectively; P < 2e–16 in all cases). Dashed black lines show the linear relationship for downregulated genes ($\log_2$ fold change < 0 at both timepoints; Pearson's correlation R = 0.50, 0.50, 0.39, 0.42, 0.26 and 0.40 for *A. vaga* and *A. ricciae*, timepoints T7 and T24 respectively; P < 2e–16 in all cases). For example, of 1093 genes that were significantly upregulated in *A. vaga* at T24, 552 (50.5%) shared an ortholog that was also significantly upregulated in *A. ricciae*, and the magnitude of DE between these orthologs was significantly correlated (Pearson's correlation R = 0.62, P < 2e–16).

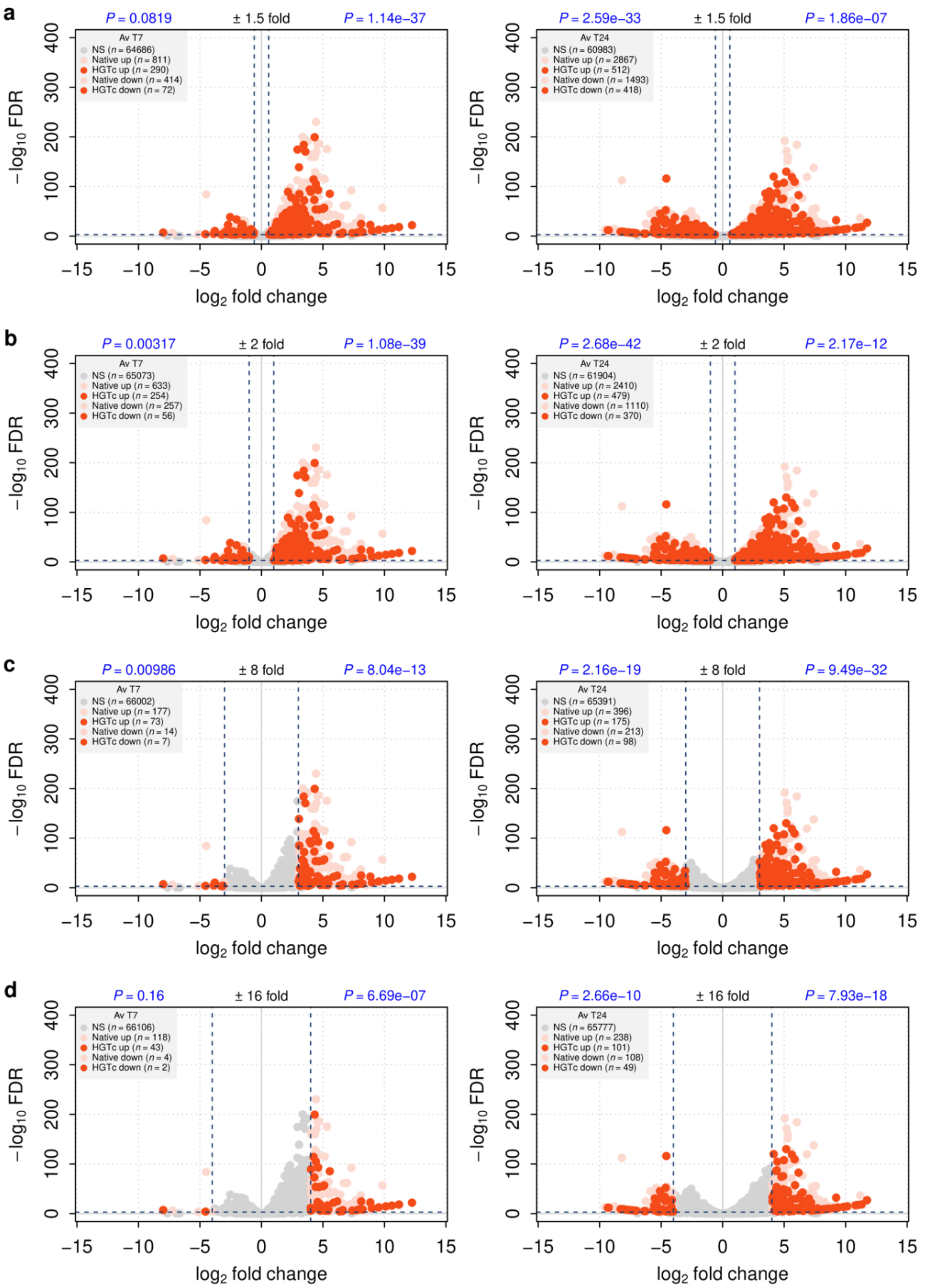

Supplementary Fig. 5. Effect of varying DE significance parameters on HGT$_C$ enrichment in *A. vaga*. Dashed lines show ± $\log_2$ fold change thresholds that are equivalent to demarking up- and downregulated subsets using **a** 1.5-fold, **b** 2-fold, **c** 8-fold, and **d** 16-fold changes in expression value. FDR threshold < 1e–3 in all cases. *P*-values in blue show the probability of observing these data given the null hypothesis that HGT$_C$ were not enriched in the corresponding subset (Fisher exact tests, two-sided).

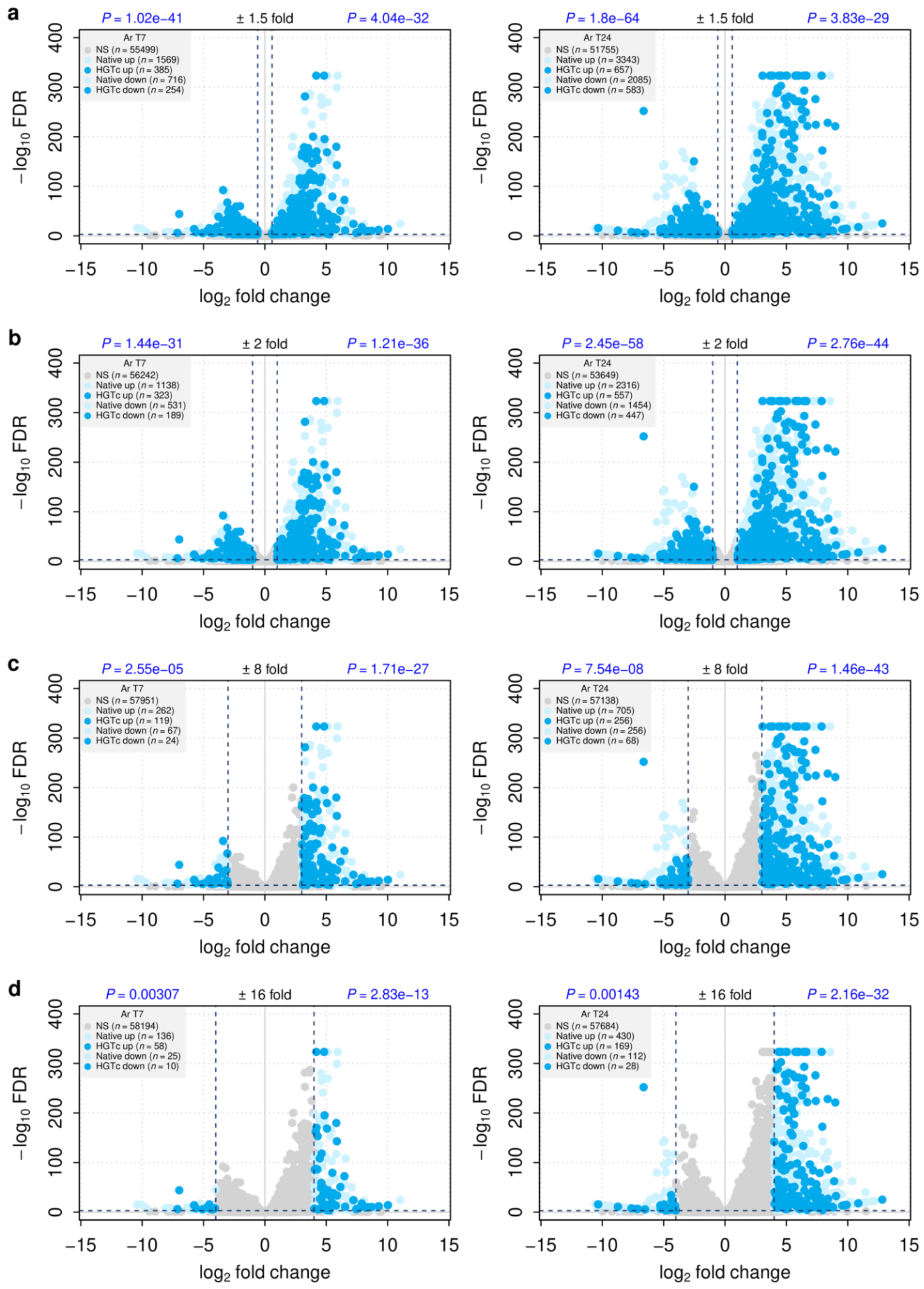

Supplementary Fig. 6. Effect of varying DE significance parameters on HGT$_C$ enrichment in *A. ricciae*. Dashed lines show ± $\log_2$ fold change thresholds that are equivalent to demarking up- and downregulated subsets using **a** 1.5-fold, **b** 2-fold, **c** 8-fold, and **d** 16-fold changes in expression value. FDR threshold < 1e–3 in all cases. *P*-values in blue show the probability of observing these data given the null hypothesis that HGT$_C$ were not enriched in the corresponding subset (Fisher exact tests, two-sided).

***A. vaga* DE results (edgeR)**

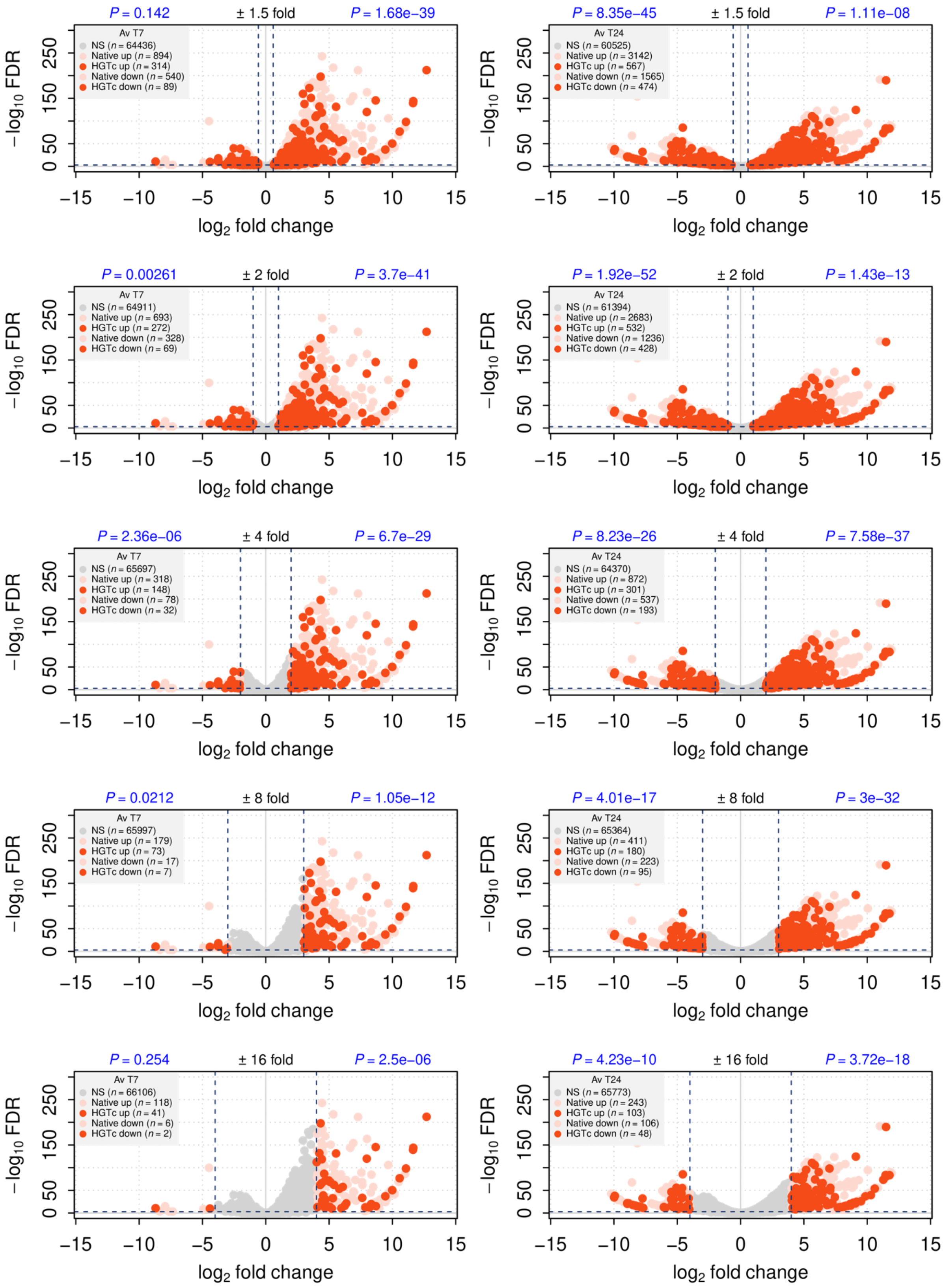

Supplementary Fig. 7. DE results using the edgeR package in *A. vaga*. Plots arranged as above.

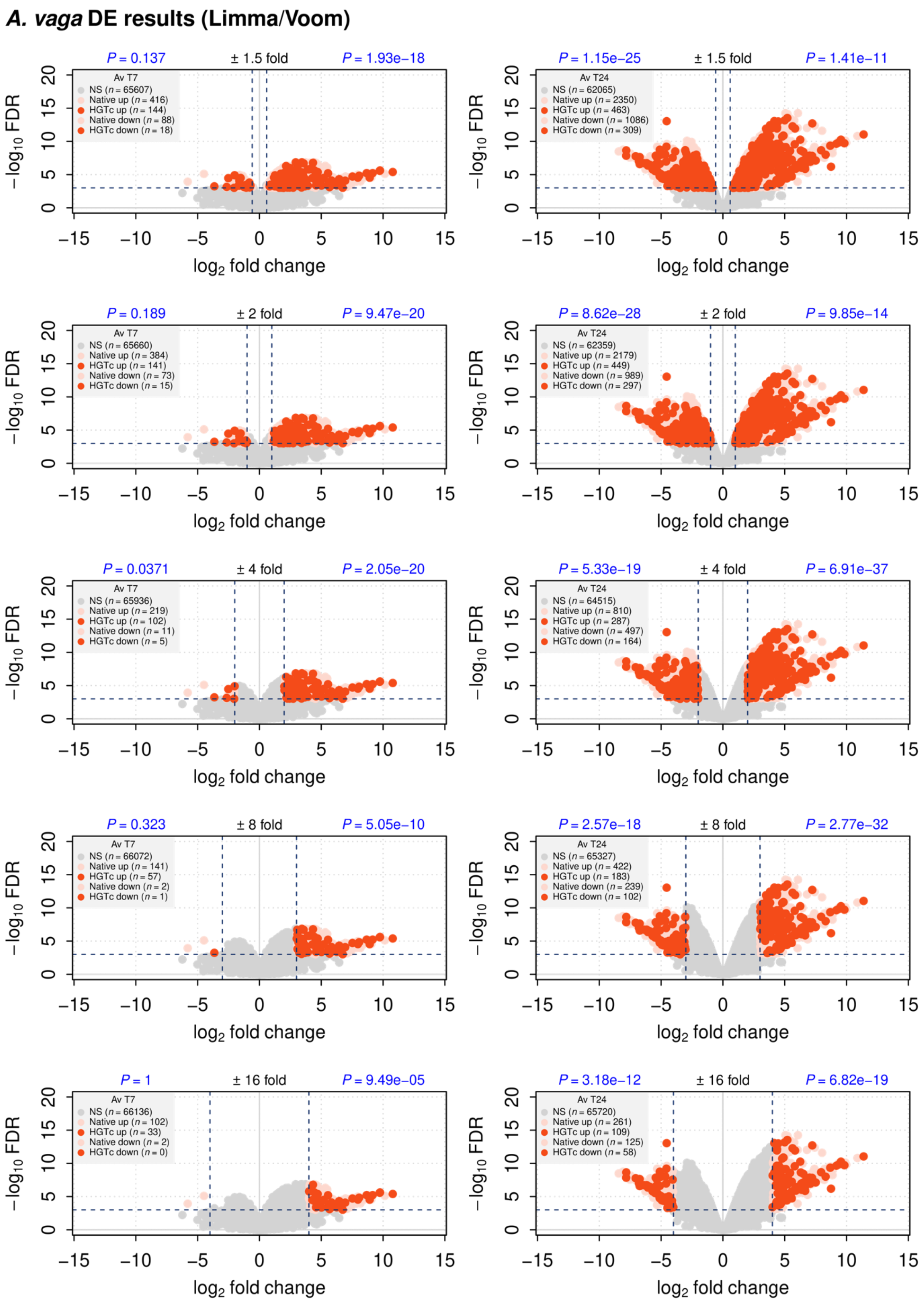

Supplementary Fig. 8. DE results using the voom package in *A. vaga*. Plots arranged as above.

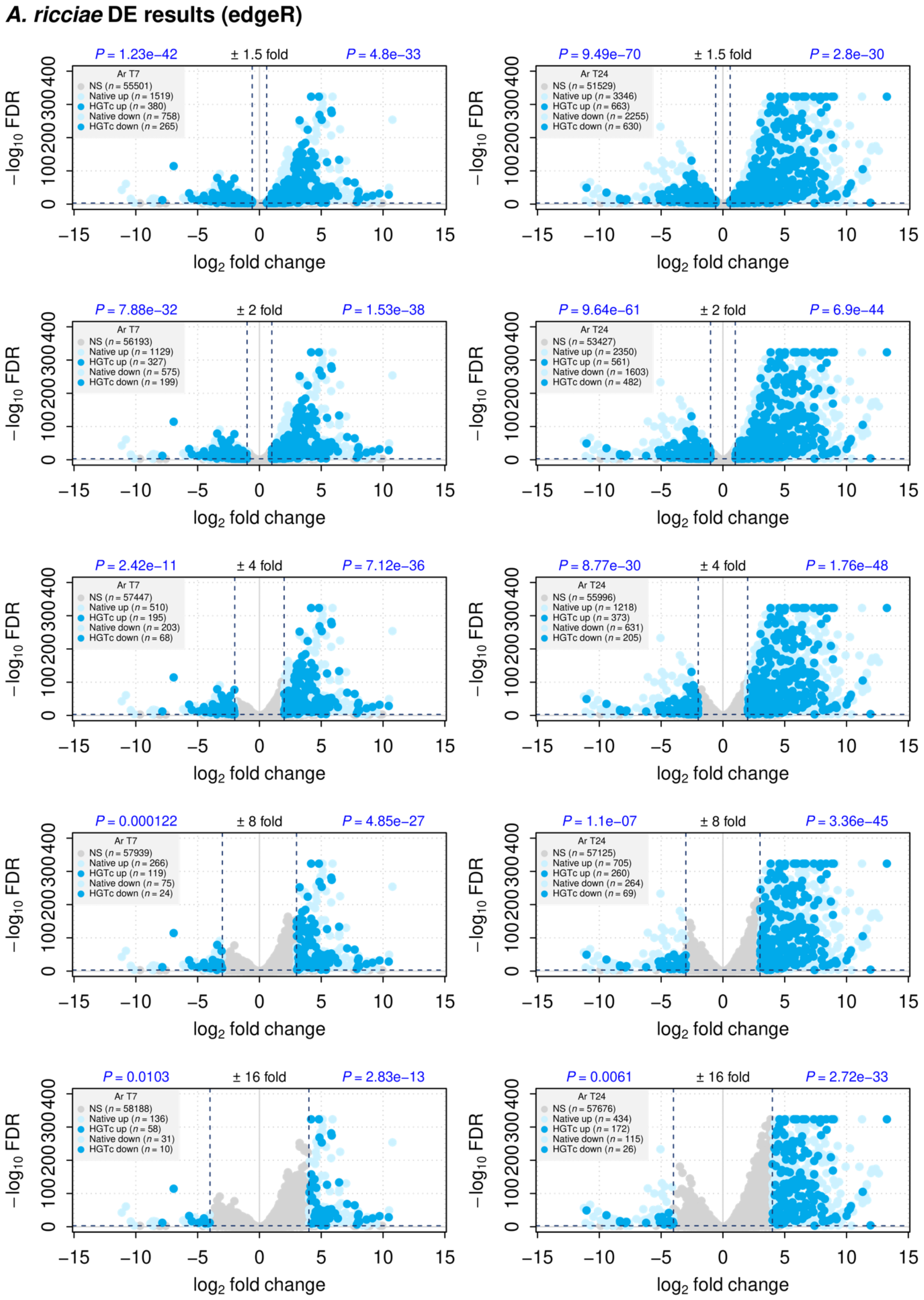

Supplementary Fig. 9. DE results using the edgeR package in *A. ricciae*. Plots arranged as above.

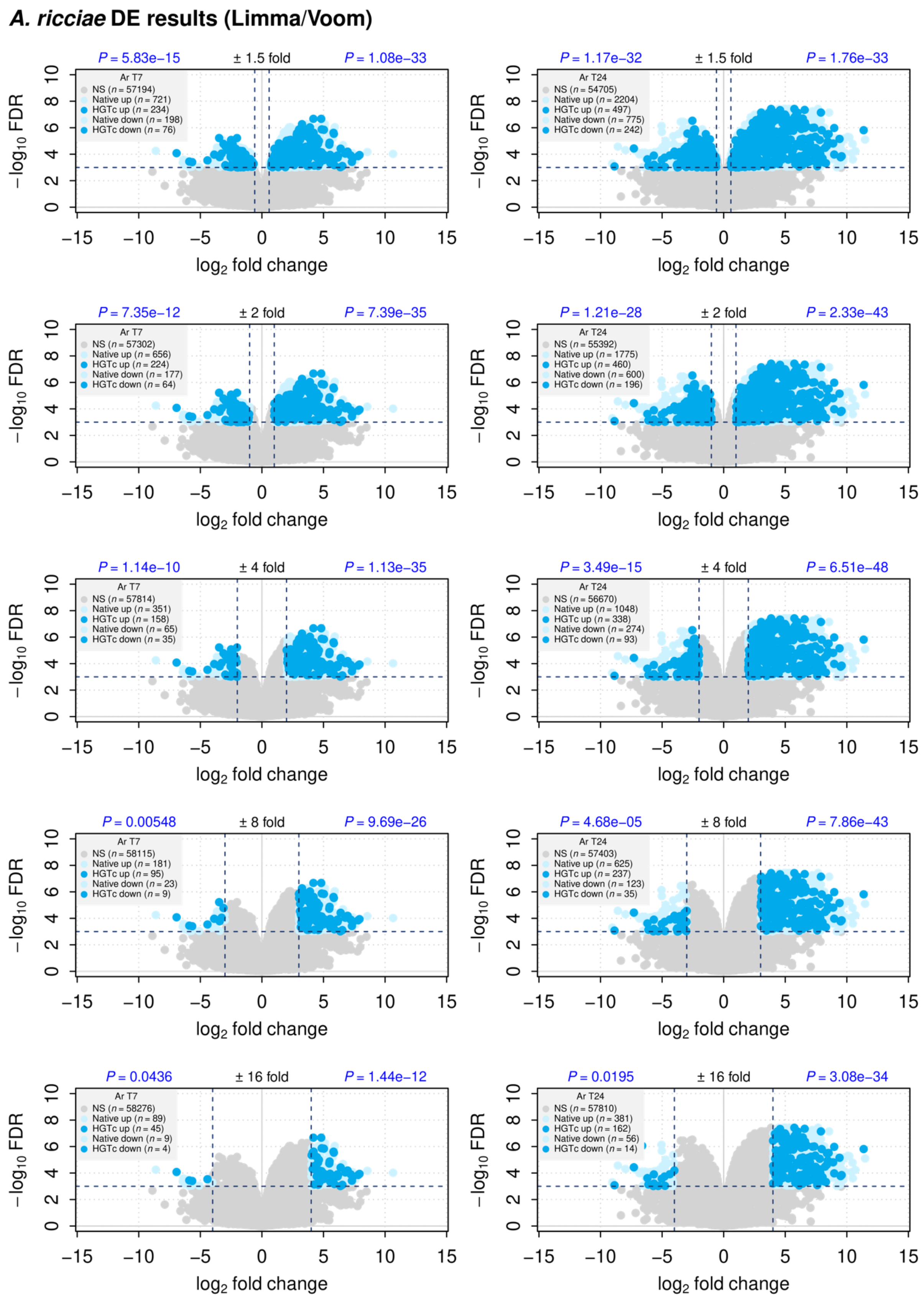

Supplementary Fig. 10. DE results using the voom package in *A. ricciae*. Plots arranged as above.

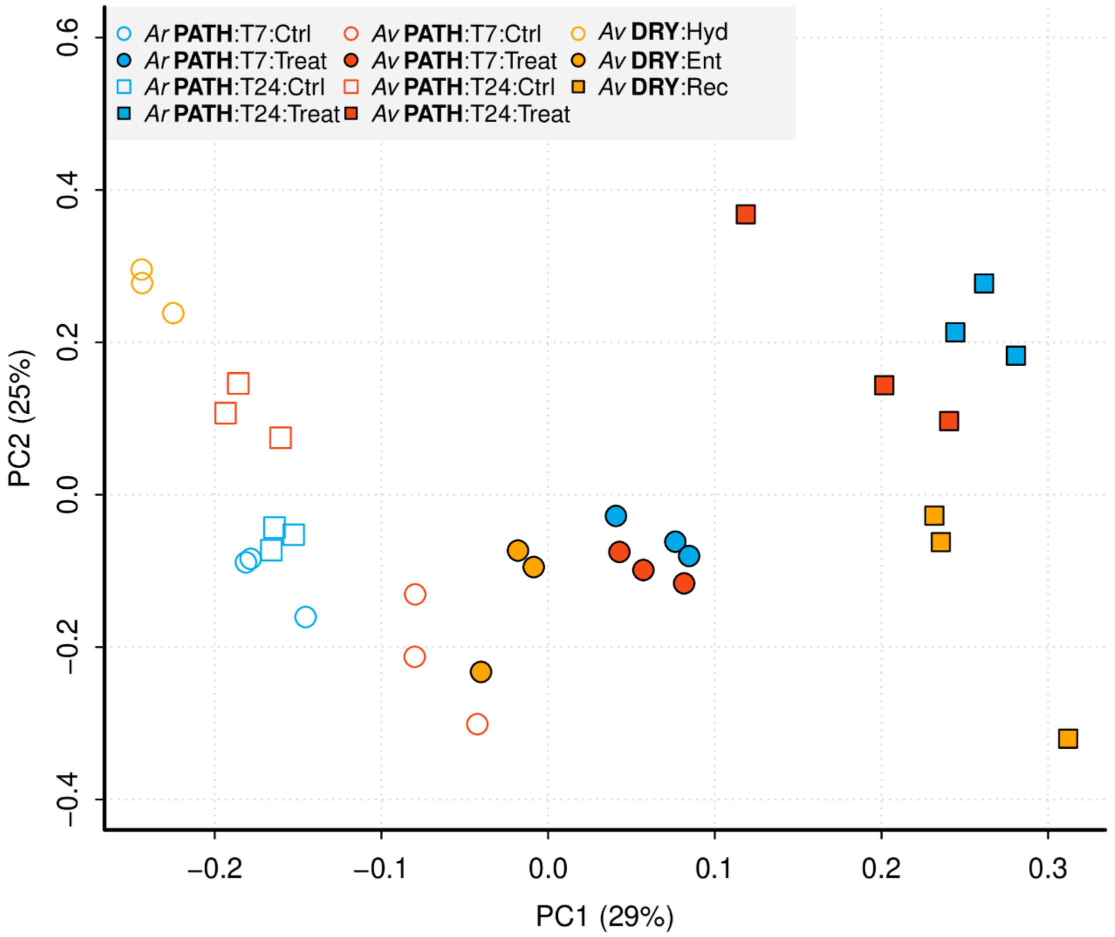

Supplementary Fig. 11. Combined PCA summarising overall patterns of gene expression across all replicates of the pathogen and desiccation stress experiments. Clustering of replicates within each treatment group is generally highly consistent. The position of replicates along the primary axis (PC1) is correlated with the relative onset and progression of the stressor in the respective experiments: ‘control’ (hydrated + uninfected); ‘early-stage stress’ (T7 + entering) and ‘later-stage stress’ (T24 + recovering). The variation among control groups within the pathogen experiment (n = 12) is similar in magnitude to the difference between the pathogen and the desiccation control groups. At the broad scale analysed here, this suggests that the baseline transcriptional state of the animals was not dramatically different between experiments, relative to variation within experiments and to the changes induced by the respective stressors.

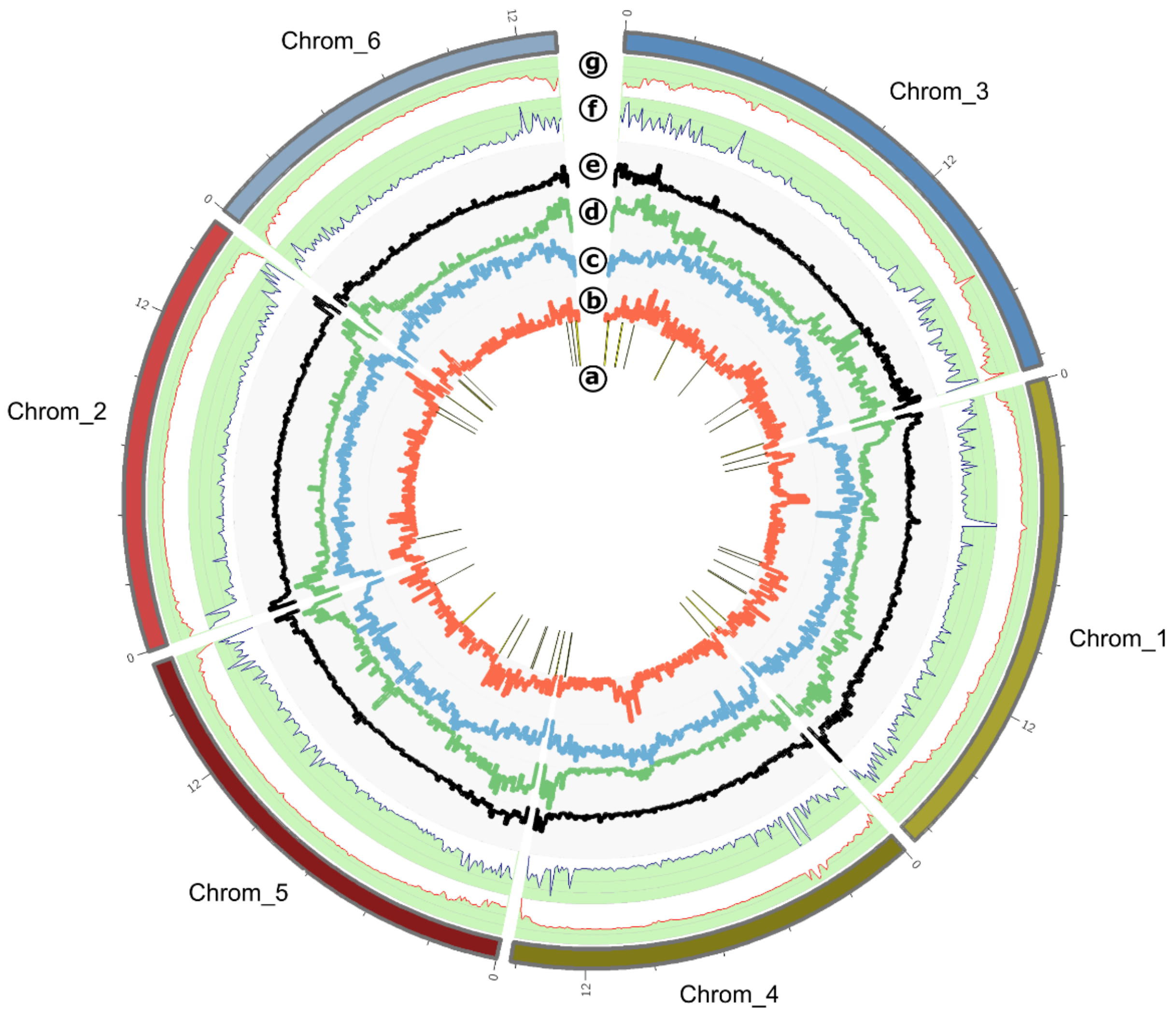

Supplementary Fig. 12. Locations of TEs, genes, HGT_C and putative secondary metabolite synthesis clusters in the Av20 haploid assembly. Profiles for chromosomes 1–6 show seven tracks: **a** biosynthetic gene clusters (black ticks with yellow highlights to indicate span), **b** HGT_C, **c** core metazoan genes, **d** TE-like elements, **e** Av13-TE consensus annotations (black histogram), **f** PacBio DNA sequencing coverage and **g** Illumina DNA sequencing coverage (red line). Transposable element (TE) repeats described by Simion et al.[47] were predominantly located in subtelomeric regions (green histogram), with distribution confirmed here by mapping compiled *A. vaga* TE consensus sequences[7] to the Av20 assembly (black histogram). Gene-rich regions (blue histogram) are mainly located outside of subtelomeric regions. Chromosome ideograms (outer layer) are plotted as colour bars (homologous pairs showing similar colour), with labels (1–6) showing chromosome numbers from the source genome assembly (GCA_021613535.1). External label ticks are spaced at 2.4 Mb apart. Coverage and histogram layers were calculated with a 100 kb sliding window. Tracks a and e are new, the others are drawn after Simion et al.[47]. Of approximately 40 putative biosynthesis clusters, two appear to be 3'-incomplete and 10 appear to contain frameshifts and/or stop codons; nevertheless, several of these are substantially transcribed.

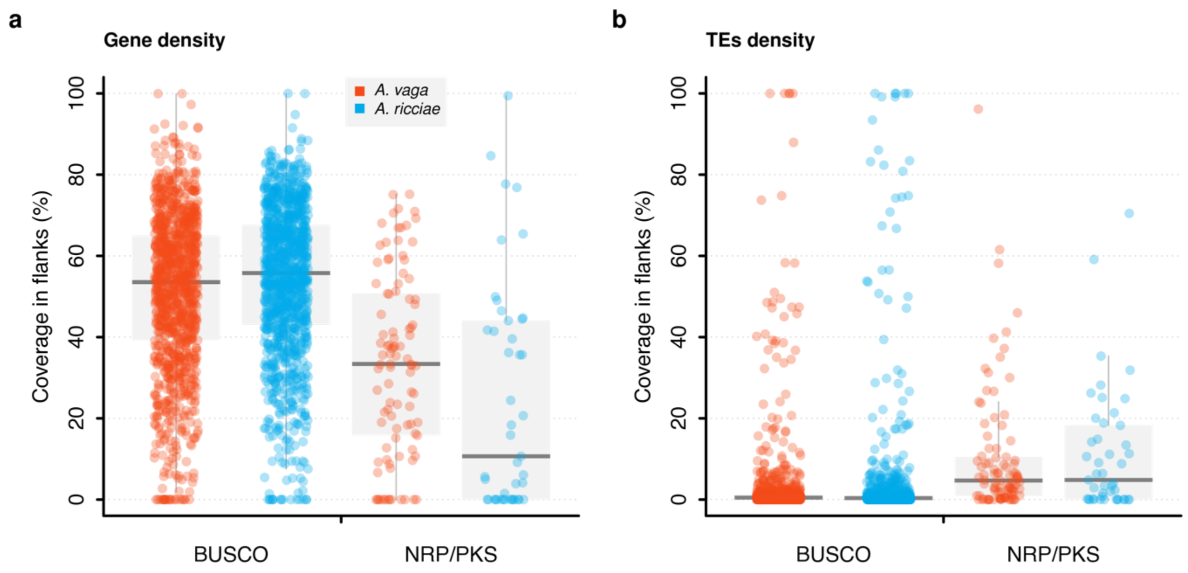

Supplementary Fig. 13. Genomic context of putative NRP/PKS clusters. The 50 kb flanking regions surrounding putative NRP/PKS gene models in both *A. vaga* and *A. ricciae* show significantly **a** fewer genes, and **b** more TEs compared to BUSCO genes (see also Fig. 5d and Supplementary Table 6 for statistical testing). *N* = 1120, 1137, 97 and 45 for *A. vaga* and *A. ricciae* BUSCO and NRP/PKS categories, respectively. Source data are provided as a Source Data file.

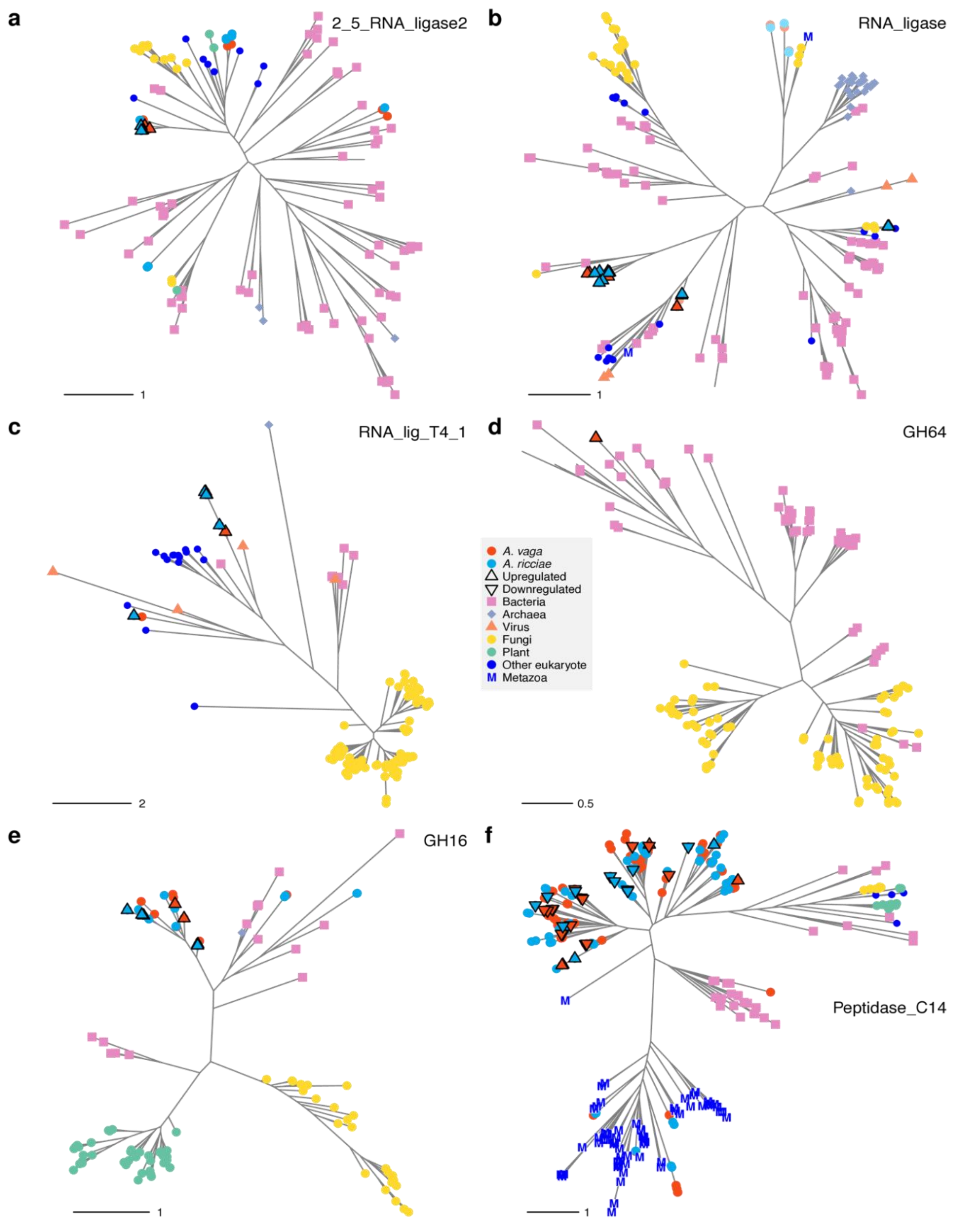

Supplementary Fig. 14. Phylogenetic relationships of bdelloid HGT$_C$ with putative roles in pathogen resistance. Trees are shown for RNA ligase domains **a** 2_5_RNA_ligase2, **b** RNA_ligase and **c** RNA_lig_T4_1; glycosyl hydrolase domains **d** Glyco_hydro_16 and **e** Glyco_hydro_64, and a caspase domain **f** Peptidase_C14. Sequences from *A. vaga* (red) and *A. ricciae* (blue) that were significantly up- or downregulated at T24 after pathogen exposure are shown as black triangles. Non-rotifer sequences were taken from the Pfam seed alignments for each domain, and thus represent the known diversity across the tree of life. Tip symbols indicate taxonomy (see legend). Metazoan sequences are indicated with a blue 'M' if present.

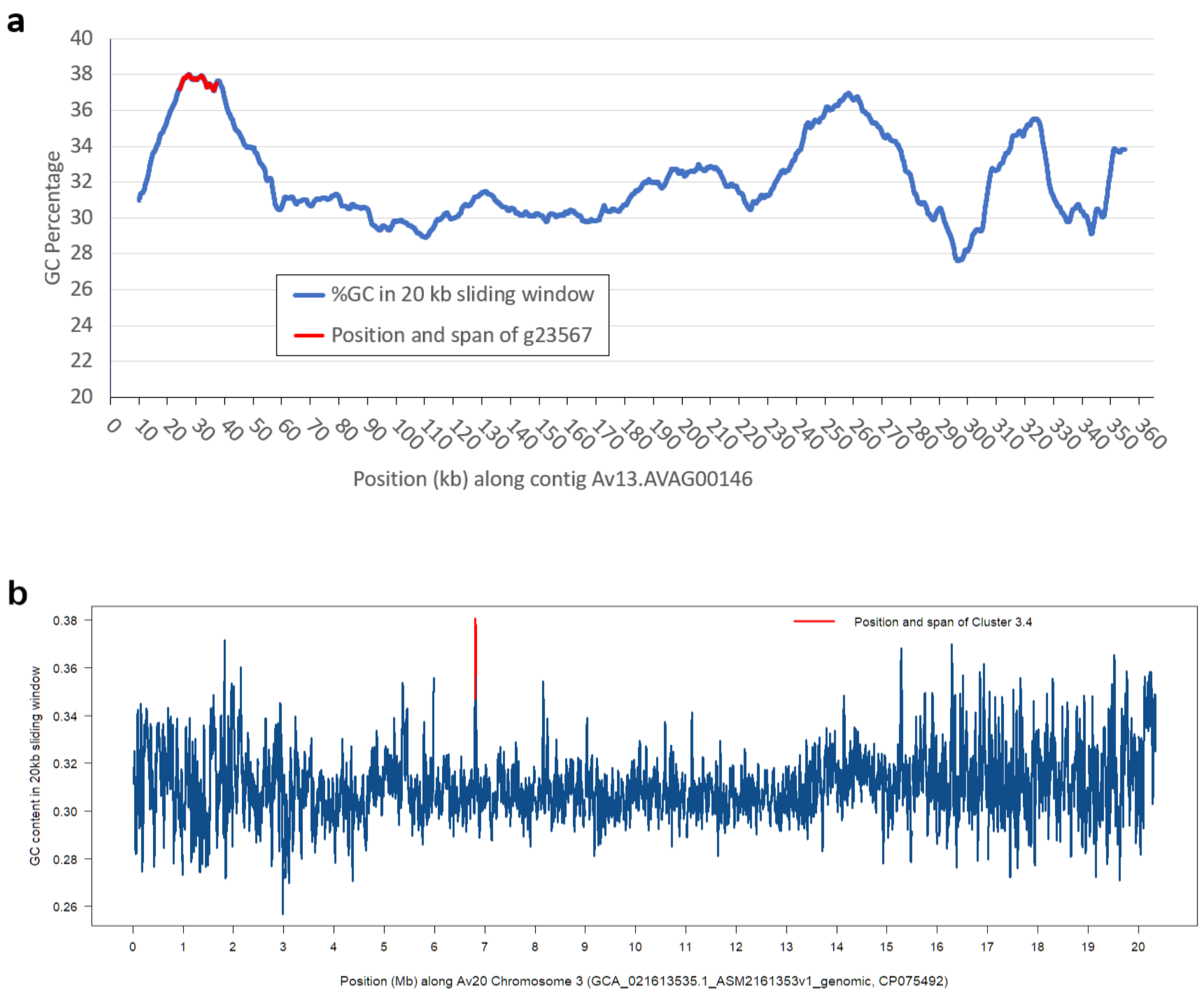

Supplementary Fig. 15. GC content surrounding the upregulated NRPS/PKS cluster corresponding to AVAG|g23567. GC content was calculated in a 20 kb sliding window along **a** contig AVAG00146 (Av13, 36.5 kb), and **b** contig CP075492 (Av20, Chromosome 3, 20.35 Mb). In each case the position of the focal annotation (shown in red) corresponds to a global peak in GC content, as predicted if this cluster had recently been acquired from a genome with a substantially higher baseline GC content than *A. vaga*.

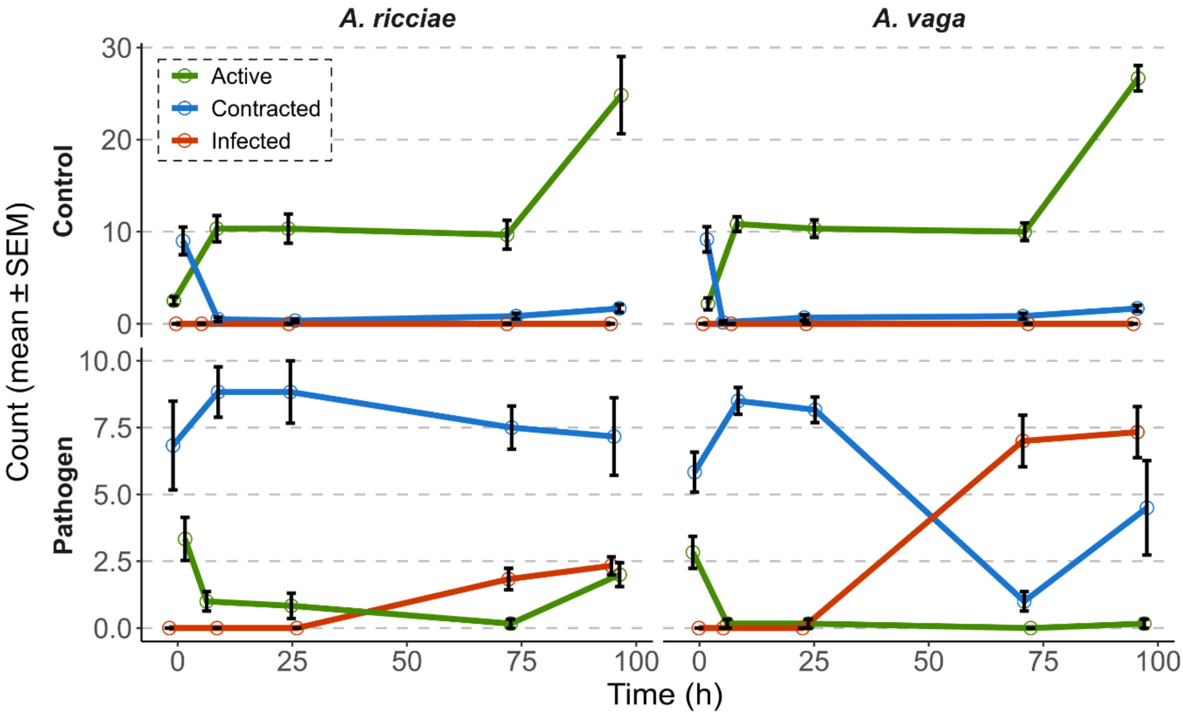

Supplementary Fig. 16. Counts of rotifers in the subset of experimental well plates coupled directly to RNA extraction tubes. Each datapoint represents six biologically replicated exposures to either live or UV-inactivated pathogen spores for *A. ricciae* (total n = 125 individuals) and *A. vaga* (n = 119). The pulse of live or inactivated spores was applied at T = 0. Between 72 and 96 hours, hatchlings began to emerge in the wells that had not been present when the spores were applied, and the infection mortality rate for the exposed cohort appeared to have stabilised. Animals dying of causes other than fungal infection are not plotted here, but this background mortality rate was < 2% of individuals. Source data are provided as a Source Data file.

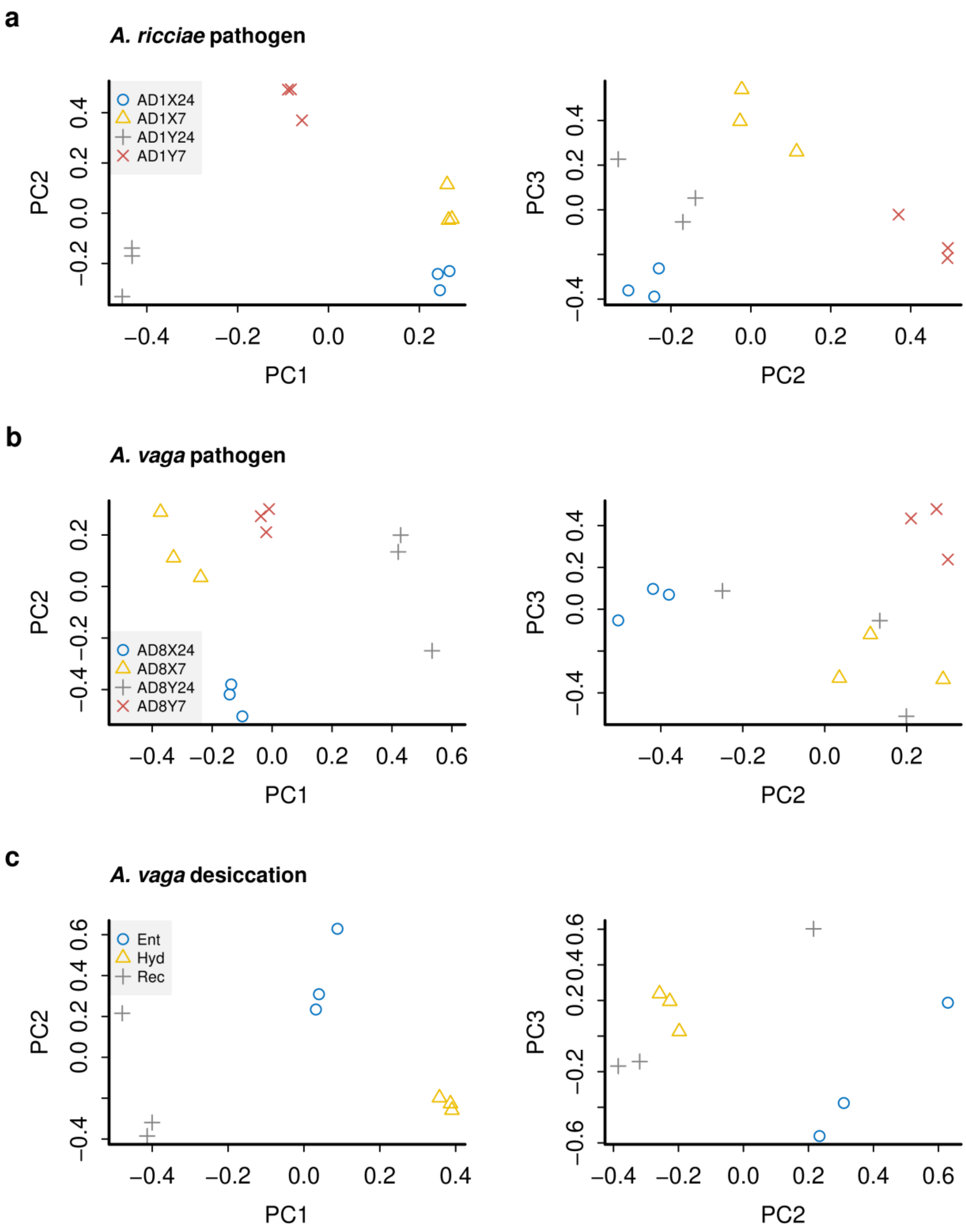

Supplementary Fig. 17. PCA of gene expression within and between biological replicates. Plots of primary versus secondary (left panels) and secondary versus tertiary (right panels) axes of variation are shown for all replicate groups of the **a** *A. ricciae* pathogen experiment, **b** *A. vaga* pathogen experiment, and **c** *A. vaga* desiccation experiment. Group codes are as follows: 'AD1' = *A. ricciae*; 'AD8' = *A. vaga*; 'X' = control; 'Y' = treatment (pathogen exposure); '7' = timepoint T7; '24' = timepoint T24; 'Ent' = entering desiccation; 'Hyd' = hydrated; 'Rec' = recovering from desiccation.

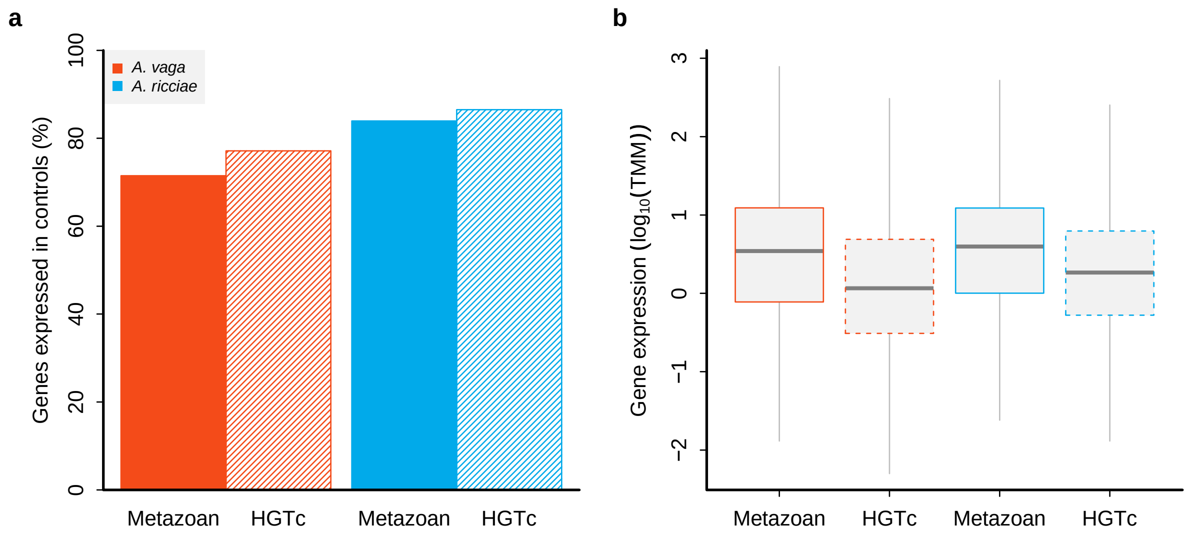

Supplementary Fig. 18. Expression of HGT$_C$ in control groups. a Proportion of genes expressed in control groups (i.e., trimmed mean of M-values [TMM] > 0 in any control replicate) by HGT$_C$ classification, for the pathogen experiment. Fisher exact test for count data, for *A. vaga* odds ratio = 1.34 (95% CI = 1.32–1.38), for *A. ricciae* odds ratio = 1.22 (95% CI = 1.19–1.27); $P < 2e{-}16$ in both cases. **b** Level of gene expression ($\log_{10}$ TMM) by HGT$_C$ classification in control replicates. Mean ($\log_{10}$) TMM *A. vaga* non-HGT$_C$ = 1.35 ± 2.38 SD, *A. vaga* HGT$_C$ = 1.03 ± 1.97 SD; Welch two sample *t*-test, $t = 80.5$ (49,628 d.f.), $P < 2e{-}16$; Mean ($\log_{10}$) TMM *A. ricciae* non-HGT$_C$ = 1.30 ± 2.31 SD; *A. ricciae* HGT$_C$ = 1.12 ± 2.08 SD; Welch two sample *t*-test, $t = 60.3$ (31,226 d.f.), $P < 2e{-}16$. N = 248,974, 37,407, 262,095 and 32,695 across categories as shown, for both panels. Source data are provided as a Source Data file.

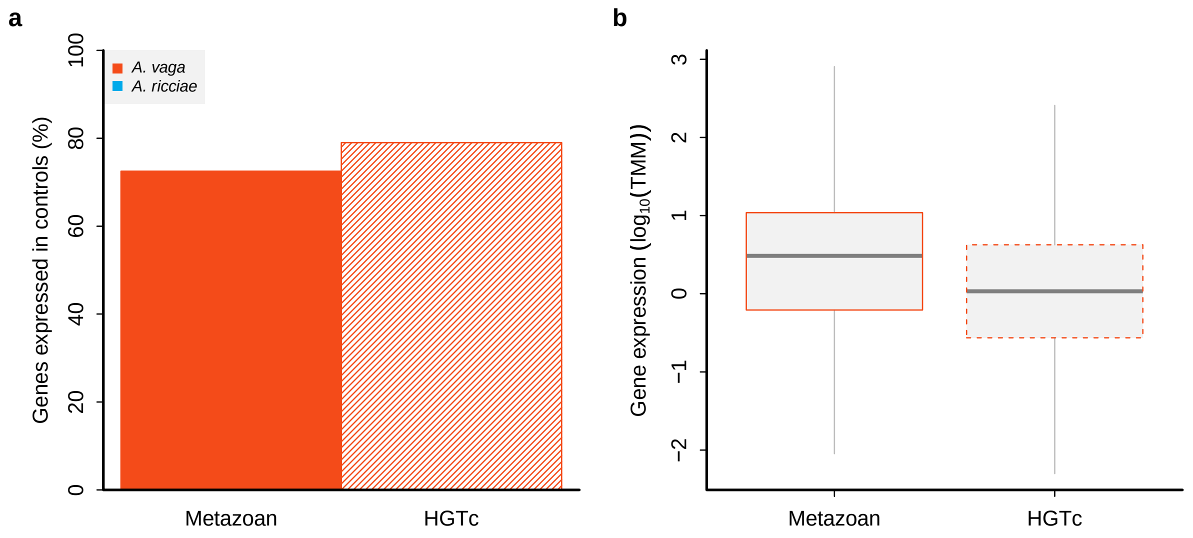

Supplementary Fig. 19. Gene expression in control replicates of *A. vaga* in a desiccation experiment. a Proportion of genes expressed in control groups (i.e., trimmed mean of M-values [TMM] > 0 in any control replicate) by HGT_C classification, for the desiccation experiment. Fisher exact test for count data, odds ratio = 1.43 (95% CI = 1.38–1.47), $P < 2e–16$. **b** Level of expression (log_{10} TMM) of HGT_C and metazoan genes expressed in control replicates. Mean (log_{10}) TMM *A. vaga* metazoan = 1.33 ± 2.57 SD; *A. vaga* HGT_C = 1.10 ± 2.27 SD; Welch two sample *t*-test, $t = 56.5$ (25,801 d.f.), $P < 2e–16$. $N = 126,293$ and 19,157 across categories as shown. Source data are provided as a Source Data file.

Supplementary Tables

Supplementary Table 1. Fixed and random effects for a linear mixed effects model testing for significant differences in magnitude of DE (normalised log2FC) between species. The reference levels for fixed factors were as follows: 'Species' = 'Av' (*A. vaga*), 'DE category' = 'NS' (i.e. genes with no significant change in expression, defined by the thresholds stated in the main text), 'Timepoint' = 'T7'. Results are based on 249,392 observations from 124,696 genes.

**Random effects**					
Groups	Variance	Std. Dev.			
Gene ID	0.2700	0.5197			
Residual	0.5679	0.7536			
**Fixed effects**					
	Estimate	Std. Error	T-value	Pr(>|t|)	Signif. code
(Intercept)	1.95e–02	3.57e–03	5.47	4.50e–08	***
speciesAr	–1.44e–01	5.23e–03	–27.461	< 2e–16	***
DE_categoryDOWN	–2.43	9.22e–02	–26.379	< 2e–16	***
DE_categoryUP	2.35	4.16e–02	56.403	< 2e–16	***
timepointT24	1.19e–01	4.18e–03	28.47	< 2e–16	***
speciesAr:DE_DOWN	8.57e–02	1.07e–01	0.798	0.4247	
speciesAr:DE_UP	7.64e–02	5.33e–02	1.433	0.1519	
speciesAr:timeT24	–1.18e–01	6.13e–03	–19.174	< 2e–16	***
DE_DOWN:timeT24	–1.12e–01	9.57e–02	–1.171	0.2417	
DE_UP:timepointT24	–2.22e–01	4.52e–02	–4.909	9.18e–07	***
speciesAr:DE_DOWN:timeT24	1.90e–01	1.13e–01	1.678	0.0934	.
speciesAr:DE_UP:timeT24	2.34e–01	5.81e–02	4.02	5.82e–05	***

Supplementary Table 2. Pearson's product-moment correlations (two-tailed) in $\log_2$ fold-change expression.

**Test**[a]	**Species**	**Timepoint**[b]	**DE type**[c]	**Pearson's *R* (95%-CI)**	***P*-value**
**Timepoints**	*A. vaga*	Both	Up	0.84 (0.84–0.85)	< 2e–16
			Down	0.67 (0.66–0.68)	< 2e–16
	A. ricciae	Both	Up	0.82 (0.82–0.83)	< 2e–16
			Down	0.66 (0.65–0.67)	< 2e–16
**Within genomes**	*A. vaga*	T7	Up	0.43 (0.42–0.44)	< 2e–16
			Down	0.50 (0.49–0.52)	< 2e–16
		T24	Up	0.43 (0.42–0.44)	< 2e–16
			Down	0.50 (0.49–0.51)	< 2e–16
	A. ricciae	T7	Up	0.49 (0.48–0.50)	< 2e–16
			Down	0.39 (0.38–0.40)	< 2e–16
		T24	Up	0.60 (0.60–0.61)	< 2e–16
			Down	0.42 (0.41–0.43)	< 2e–16
**Between genomes**	Both	T7	Up	0.35 (0.34–0.36)	< 2e–16
			Down	0.26 (0.25–0.27)	< 2e–16
		T24	Up	0.37 (0.36–0.37)	< 2e–16
			Down	0.40 (0.39–0.40)	< 2e–16

[a]Explanation of tests: "Timepoints", correlation in differential expression (DE) in the same gene across timepoints T7 and T24 (Supplementary Fig. 2); "Within genomes", correlation in DE between inferred gene copies within genomes (including putative homologs, homoeologs and other gene copies) (Supplementary Fig. 4a–d); "Between genomes"; correlation in DE between inferred orthologs across species (Supplementary Fig. 4e–f).
[b]Timepoints: "T7", 7h post-infection; "T24", 24h post-infection.
[c]DE type: "Up", genes that are upregulated in the treatment group (i.e., positive DE); "Down", genes that are downregulated in the treatment group (negative DE). Note that all genes are included in correlations regardless of significance.

Supplementary Table 3. Statistical analysis of HGT_C enrichment among DE genes.

a Summary of a generalised (binomial) linear model testing for enrichment of HGT_C in up- and downregulated subsets of genes following exposure to a fungal pathogen. The reference levels for fixed factors were as follows: 'DE category' = 'NS' (i.e. genes with no significant change in expression, defined by the thresholds stated in the main text); 'timepoint' = 'T7'. Results are based on 249,392 observations of 124,696 genes.

	Estimate	Std. Error	Z-value	Pr(>|z|)	Signif. code
(Intercept)	–2.057368	0.008989	–228.886	<2e–16	***
DE_categoryDOWN	1.087234	0.121459	8.951	<2e–16	***
DE_categoryUP	1.179946	0.065063	18.136	<2e–16	***
timepointT24	–0.024297	0.012843	–1.892	0.0585	.
DE categoryDOWN:timeT24	–0.096948	0.135819	–0.714	0.4753	
DE categoryUP:timeT24	–0.224416	0.079581	–2.82	0.0048	**

b Fixed effects for generalized linear models run separately for timepoints T7 and T24. The reference levels for fixed factors were as follows: 'Species' = 'Av' (*A. vaga*), 'DE category' = 'NS' (i.e. genes with no significant change in expression, defined by the thresholds stated in the main text).

**Timepoint T7**					
	Estimate	Std. Error	Z-value	Pr(>|z|)	Signif. code
(Intercept)	–1.98809	0.01198	–165.911	< 2e–16	***
DE_category.DOWN	1.10305	0.23341	4.726	2.29e–06	***
DE_category.UP	1.23798	0.10148	12.200	< 2e–16	***
Species.Ar	–0.15337	0.01813	–8.461	< 2e–16	***
DE_category.DOWN:species.Ar	0.03756	0.27345	0.137	0.891	
DE_category.UP:species.Ar	–0.05868	0.13242	–0.443	0.658	
**Timepoint T24**					
(Intercept)	–2.00893	0.01219	–164.768	< 2e–16	***
DE_category.DOWN	0.96111	0.08870	10.836	< 2e–16	***
DE_category.UP	0.96686	0.06996	13.819	< 2e–16	***
Species.Ar	–0.16190	0.01851	–8.744	< 2e–16	***
DE_category.DOWN:species.Ar	0.08080	0.12185	0.663	0.507	
DE_category.UP:species.Ar	0.01790	0.09273	0.193	0.847	

Supplementary Table 4. Fixed effects for a generalized linear model testing for significant enrichment of HGT_C in up- and downregulated subsets of genes while entering and recovering from desiccation. The reference levels for the fixed factor 'DE category' = 'NS' (i.e. genes with no significant change in expression, defined by the thresholds stated in the main text).

**Entering desiccation**					
	Estimate	Std. Error	Z-value	Pr(>|z|)	Signif. code
(Intercept)	–1.97337	0.01195	–165.126	< 2e–16	***
DE_category.DOWN	–0.19646	0.17393	–1.130	0.259	
DE_category.UP	0.14130	0.12332	1.146	0.252	
**Recovering from desiccation**					
(Intercept)	–1.99608	0.01226	–162.771	< 2e–16	***
DE_category.DOWN	0.63605	0.06932	9.176	< 2e–16	***
DE_category.UP	0.23939	0.06752	3.546	3.92e–3	***

Supplementary Table 5. Proportion of *A. vaga* DE genes shared between pathogen and desiccation conditions. Values in bold on diagonals indicate the total number of genes in each category (e.g., 136 HGT_C genes upregulated at T7). Percentages in each cell then indicate the proportion of shared genes relative to the row total (e.g., 89% of 145 T7 upregulated HGT_C genes are shared with T24 upregulated set). Green and red shading is to highlight the relative size of the shared fraction for up- and downregulated subsets, respectively.

**Gene type**	**DE type**		Pathogen (T7)	Pathogen (T24)	Entering	Recovering
All DE genes	Up	T7	**452**	89.6%	7.1%	21.9%
All DE genes	Up	T24	37.1%	**1093**	9.3%	20.9%
All DE genes	Up	Ent	5.7%	18.3%	**558**	62.7%
All DE genes	Up	Rec	5.5%	12.6%	19.4%	**1807**
All DE genes	Down	T7	**89**	66.3%	2.2%	0.0%
All DE genes	Down	T24	8.8%	**674**	2.1%	11.3%
All DE genes	Down	Ent	0.6%	3.9%	**361**	26.3%
All DE genes	Down	Rec	0.0%	5.7%	7.2%	**1322**
HGT_C	Up	T7	**145**	89.0%	9.7%	22.8%
HGT_C	Up	T24	45.3%	**285**	10.9%	22.1%
HGT_C	Up	Ent	18.2%	40.3%	**77**	57.1%
HGT_C	Up	Rec	12.4%	23.7%	16.5%	**266**
HGT_C	Down	T7	**26**	76.9%	3.8%	0.0%
HGT_C	Down	T24	11.4%	**175**	2.3%	14.9%
HGT_C	Down	Ent	2.7%	10.8%	**37**	40.5%
HGT_C	Down	Rec	0.0%	9.6%	5.6%	**270**

Supplementary Table 6. Fixed and random effects for linear mixed effects models testing for an association between putative NRP/PKS genes and three genomic features of interest **a** gene density, **b** transposable elements, and **c** telomeric repeats, as measured by the proportion (%) of base pairs occupied by these features in 25 kb flanking regions (minimum = 1 kb) surrounding putative NRP/PKS genes compared to BUSCO genes.

a Gene density. The reference level for the fixed factor 'Type' = 'BUSCO'.

**Random effects**					
Groups	Variance	Std. Dev.			
SPECIES	0.000195	0.01397			
Residual	0.037318	0.19318			
**Fixed effects**					
	Estimate	Std. Error	T-value	Pr(>|t|)	Signif.
(Intercept)	0.52504	0.01068	49.16	0.0122	*
TYPE.NRPS	–0.21515	0.01677	–12.83	< 2e–16	***

b Transposable elements. The reference level for the fixed factor 'Type' = 'BUSCO'.

**Random effects**					
Groups	Variance	Std. Dev.			
Species	0.003056	0.05528			
Residual	0.426313	0.65293			
**Fixed effects**					
	Estimate	Std. Error	T-value	Pr(>|t|)	Signif.
(Intercept)	– 2.24721	0.04143	– 54.23	0.0112	*
TYPE.NRPS	0.70951	0.70951	12.52	< 2e–16	***

c Telomeric repeats. The reference level for the fixed factor 'Type' = 'BUSCO'.

**Random effects**					
Groups	Variance	Std. Dev.			
Species	0.001372	0.03704			
Residual	0.026702	0.16341			
**Fixed effects**					
	Estimate	Std. Error	T-value	Pr(>|t|)	Signif.
(Intercept)	–2.66508	0.02642	–100.88	0.00626	**
TYPE.NRPS	0.20338	0.01419	14.33	< 2e–16	***

Supplementary Table 7. Determination of flanking genes in paralogous *A. ricciae* copies of the *A. vaga* PKS-NRPS cluster AVAG|g48151 shown in Fig. 5b, using alternative 2021 assembly (Ar21[41]). Bold font denotes flanking genes resembling putative drug transporters (MFS/ABCB)[71] or encoding the cytochrome P450 domain, associated with metazoan detoxification of xenobiotic metabolites and with their biosynthesis and biotransformation in bacteria[72]. Matching Ar18 gene models are listed and the differential expression status of these flanking genes is shown in Supplementary Data 10 (all are highly upregulated together with their neighbouring NRP/PKS).

Ar18_CDS	Ar18 contig	Accession No.	Ar21 contig	Accession No.	5'-adjacent gene	3'-adjacent gene	Ar18_CDS for flanking transporter-like genes
**ARIC|g35898**	ARIC00373	OEQJ01000373.1	ARIC003_00297	CAJNOJ010000297.1	**ABC transporter B family**	**CYP (cytochrome P450)**	ARIC|g35900; ARIC|g35896
**ARIC|g15363**	ARIC00114	OEQJ01000114.1	ARIC003_00085	CAJNOJ010000085.1	ASC (amiloride-sensitive Na+ channel)	n/a	
**ARIC|g49019**	ARIC01129	OEQJ01001129.1	ARIC003_00110	CAJNOJ010000110.1	n/a	RRM1_CPEB2-like family protein	
**ARIC|g51868**	ARIC01782	OEQJ01001782.1	ARIC003_01423	CAJNOR010001423.1	**MFS transporter-like protein**	n/a	ARIC|g48114; ARIC|g51163
**ARIC|g51257**	ARIC01461	OEQJ01001461.1	ARIC003_01015	CAJNOJ010001015.1	n/a	**ABC transporter B family**	ARIC|g48115; ARIC|g48116

Supplementary Table 8. Evidence for the hypothesis that upregulation of HGT_C-enriched effectors might be paired with downregulation of HGT_C-enriched negative regulators or repressors. Occurrence of the word "regulation" in the text of GO terms that are significantly enriched among differentially expressed HGT_C genes. This word appears only in terms enriched in downregulated genes, and at a significantly higher rate in the pathogen response than in the desiccation response, where HGT_C are not significantly enriched. For instance, it only appears once among the 55 GO terms enriched among HGT_C that are downregulated during desiccation recovery, where there was correspondingly only very weak enrichment of upregulated HGT_C (Fig. 3b). Fisher's exact test for frequency of "regulation" in *A. vaga* pathogen T24 downregulated versus desiccation recovery downregulated terms: $P < 0.0001$.

	A. ricciae				***A. vaga***					
	Pathogen T7		Pathogen T24		Pathogen T7		Pathogen T24		Desiccation recovery	
	UP	**DOWN**	UP	**DOWN**	UP	**DOWN**	UP	**DOWN**	UP	**DOWN**
**"regulation" in term**	0	14	0	13	0	18	0	18	0	1
**Total enriched GO terms (FDR<0.001)**	15	28	29	33	8	41	18	59	4	55
**Proportion of terms**	0	**0.5**	0	**0.39**	0	**0.44**	0	**0.31**	0	0.02

Supplementary Table 9. Data counts for raw and filtered sequencing data.

Sample ID	Species	Group	Reads (raw)	Reads (trimmed)	Bases (trimmed)	Reads (filtered)	Bases (filtered)	% (raw)	SRA accession
AD1X24b	*Adineta ricciae*	24h control	72,300,282	71,461,358	3,572,336,554	66,227,634	3,310,716,200	91.6%	ERR4469902
AD1X24c	*Adineta ricciae*	24h control	103,152,802	101,927,044	5,095,297,800	94,024,076	4,700,243,436	91.1%	ERR4471101
AD1X24d	*Adineta ricciae*	24h control	80,035,292	78,926,744	3,945,535,594	76,229,980	3,810,727,560	95.2%	ERR4469904
AD1X7a	*Adineta ricciae*	7h control	110,227,526	109,122,278	5,455,025,296	101,914,574	5,094,710,998	92.4%	ERR4471102
AD1X7b	*Adineta ricciae*	7h control	77,501,642	76,710,200	3,834,748,236	71,677,976	3,583,188,364	92.5%	ERR4469903
AD1X7c	*Adineta ricciae*	7h control	95,649,472	94,620,052	4,730,073,808	88,264,972	4,412,373,744	92.3%	ERR4469908
AD1Y24b	*Adineta ricciae*	24h treatment	80,992,896	79,874,140	3,992,916,338	72,107,000	3,604,641,298	89.0%	ERR4469905
AD1Y24c	*Adineta ricciae*	24h treatment	92,458,758	91,140,052	4,556,094,802	84,864,356	4,242,376,646	91.8%	ERR4469907
AD1Y24d	*Adineta ricciae*	24h treatment	73,779,290	72,716,918	3,635,125,000	68,816,850	3,440,163,964	93.3%	ERR4469891
AD1Y7a	*Adineta ricciae*	7h treatment	95,898,178	94,647,472	4,731,446,522	88,073,474	4,402,804,314	91.8%	ERR4471099
AD1Y7c	*Adineta ricciae*	7h treatment	80,801,710	79,603,168	3,979,351,112	59,626,746	2,980,729,936	73.8%	ERR4469906
AD1Y7d	*Adineta ricciae*	7h treatment	106,034,116	104,635,518	5,230,757,386	91,899,730	4,594,073,480	86.7%	ERR4471100
AD8X24b	*Adineta vaga*	24h control	77,324,552	76,491,012	3,823,681,760	72,241,176	3,611,252,230	93.4%	ERR4471106
AD8X24c	*Adineta vaga*	24h control	97,572,802	96,602,138	4,829,003,374	88,430,872	4,420,542,982	90.6%	ERR4471110
AD8X24d	*Adineta vaga*	24h control	81,497,256	80,569,634	4,027,596,446	75,066,308	3,752,507,226	92.1%	ERR4471109
AD8X7a	*Adineta vaga*	7h control	107,985,090	106,534,822	5,325,496,126	100,763,764	5,037,013,014	93.3%	ERR4471115
AD8X7b	*Adineta vaga*	7h control	93,504,888	92,462,058	4,622,044,962	80,579,888	4,028,031,738	86.2%	ERR4471114
AD8X7c	*Adineta vaga*	7h control	82,552,616	81,515,658	4,074,864,246	77,159,840	3,857,128,480	93.4%	ERR4471113
AD8Y24a	*Adineta vaga*	24h treatment	70,766,208	69,666,792	3,482,567,840	66,005,632	3,299,557,790	93.3%	ERR4471108
AD8Y24b	*Adineta vaga*	24h treatment	76,829,912	75,812,588	3,789,768,588	72,408,734	3,619,621,438	94.2%	ERR4471104
AD8Y24c	*Adineta vaga*	24h treatment	70,353,904	69,337,914	3,466,123,464	64,429,388	3,220,751,222	91.6%	ERR4471111
AD8Y7a	*Adineta vaga*	7h treatment	105,228,144	103,919,566	5,194,775,380	97,013,708	4,849,556,092	92.2%	ERR4471116
AD8Y7c	*Adineta vaga*	7h treatment	78,242,264	77,242,564	3,861,269,744	64,664,014	3,232,464,688	82.6%	ERR4471105
AD8Y7d	*Adineta vaga*	7h treatment	72,992,040	72,083,380	3,603,389,336	61,679,400	3,083,272,030	84.5%	ERR4471107

Supplementary Table 10. Assembly and quality metrics for Trinity *de novo* transcriptomes.

	A. ricciae	***A. vaga***
**Number of transcripts**	118,860	63,749
**Mean**	1,400	1,396
**Median**	1,157	1,080
**Max**	31,021	20,120
**Total span**	166,388,199	88,983,009
**GC%**	37.6	33.0
**Number of 'genes'**	41,527	35,079
**Total span of 'genes'**	48,809,330	41,442,308
**BUSCO[a] eukaryote (*n* = 303)**	C:97.7%[S:10.9%,D:86.8%],F:1.7%	C:99.0%[S:57.4%,D:41.6%],F:0.7%
**BUSCO metazoa (*n* = 978)**	C:93.4%[S:11.5%,D:81.9%],F:1.4%	C:93.8%[S:51.8%,D:42.0%],F:1.0%
**Mapped reads to transcriptome**	99.4%	99.5%
**Mapped transcripts to genome[b]**	118,277 (99.5%)	63,379 (99.4%)
**Introns predicted from GFF**	478,936	276,708
**Correct introns from mapping**	417,174 (87.1%)	226,332 (81.8%)

[a]BUSCO codes: **C**, complete; **S**, complete and single copy; **D**, complete and duplicated; **F**, fragmented.
[b]GenBank accessions: *A. ricciae*, GCA_900240375.1; *A. vaga*, GCA_000513175.1.

Supplementary Table 11. Proportion of raw RNA-seq data mapping to *E. coli* OP50 genome.

Sample ID	Species	Total reads	Mapped reads	%	Total bases	Mapped bases	%
AD1X24b	*Adineta ricciae*	72300282	920490	1.273%	3615014100	46024500	1.273%
AD1X24c	*Adineta ricciae*	103152802	1471481	1.427%	5157640100	73574050	1.427%
AD1X24d	*Adineta ricciae*	80035292	399803	0.500%	4001764600	19990150	0.500%
AD1X7a	*Adineta ricciae*	110227526	2457820	2.230%	5511376300	122891000	2.230%
AD1X7b	*Adineta ricciae*	77501642	1429477	1.844%	3875082100	71473850	1.844%
AD1X7c	*Adineta ricciae*	95649472	2498017	2.612%	4782473600	124900850	2.612%
AD1Y24b	*Adineta ricciae*	80992896	2477221	3.059%	4049644800	123861050	3.059%
AD1Y24c	*Adineta ricciae*	92458758	2122877	2.296%	4622937900	106143850	2.296%
AD1Y24d	*Adineta ricciae*	73779290	1234554	1.673%	3688964500	61727700	1.673%
AD1Y7a	*Adineta ricciae*	95898178	2406328	2.509%	4794908900	120316400	2.509%
AD1Y7c	*Adineta ricciae*	80801710	9639335	11.930%	4040085500	481966750	11.930%
AD1Y7d	*Adineta ricciae*	106034116	5739959	5.413%	5301705800	286997950	5.413%
AD8X24b	*Adineta vaga*	77324552	1170726	1.514%	3866227600	58536300	1.514%
AD8X24c	*Adineta vaga*	97572802	2515106	2.578%	4878640100	125755300	2.578%
AD8X24d	*Adineta vaga*	81497256	1780058	2.184%	4074862800	89002900	2.184%
AD8X7a	*Adineta vaga*	107985090	3040177	2.815%	5399254500	152008850	2.815%
AD8X7b	*Adineta vaga*	93504888	7089717	7.582%	4675244400	354485850	7.582%
AD8X7c	*Adineta vaga*	82552616	2237871	2.711%	4127630800	111893550	2.711%
AD8Y24a	*Adineta vaga*	70766208	1452059	2.052%	3538310400	72602950	2.052%
AD8Y24b	*Adineta vaga*	76829912	1503493	1.957%	3841495600	75174650	1.957%
AD8Y24c	*Adineta vaga*	70353904	2737599	3.891%	3517695200	136879950	3.891%
AD8Y7a	*Adineta vaga*	105228144	3860181	3.668%	5261407200	193009050	3.668%
AD8Y7c	*Adineta vaga*	78242264	7091724	9.064%	3912113200	354586200	9.064%
AD8Y7d	*Adineta vaga*	72992040	6245464	8.556%	3649602000	312273200	8.556%

Supplementary Table 12. Proportion of filtered RNA-seq data mapping to *E. coli* OP50 genome.

Sample ID	Species	Total reads	Mapped reads	%	Total bases	Mapped bases	%
AD1X24b	*Adineta ricciae*	66227634	2540	0.004%	3310716200	126964	0.004%
AD1X24c	*Adineta ricciae*	94024076	3869	0.004%	4700243436	193427	0.004%
AD1X24d	*Adineta ricciae*	76229980	1984	0.003%	3810727560	99189	0.003%
AD1X7a	*Adineta ricciae*	101914574	8800	0.009%	5094710998	439925	0.009%
AD1X7b	*Adineta ricciae*	71677976	4079	0.006%	3583188364	203939	0.006%
AD1X7c	*Adineta ricciae*	88264972	8537	0.010%	4412373744	426824	0.010%
AD1Y24b	*Adineta ricciae*	72107000	6247	0.009%	3604641298	312326	0.009%
AD1Y24c	*Adineta ricciae*	84864356	6671	0.008%	4242376646	333452	0.008%
AD1Y24d	*Adineta ricciae*	68816850	4148	0.006%	3440163964	207340	0.006%
AD1Y7a	*Adineta ricciae*	88073474	7420	0.008%	4402804314	370968	0.008%
AD1Y7c	*Adineta ricciae*	59626746	37248	0.063%	2980729936	1861604	0.063%
AD1Y7d	*Adineta ricciae*	91899730	15321	0.017%	4594073480	765977	0.017%
AD8X24b	*Adineta vaga*	72241176	4026	0.006%	3611252230	201274	0.006%
AD8X24c	*Adineta vaga*	88430872	8762	0.010%	4420542982	437924	0.010%
AD8X24d	*Adineta vaga*	75066308	7264	0.010%	3752507226	363134	0.010%
AD8X7a	*Adineta vaga*	100763764	16084	0.016%	5037013014	803992	0.016%
AD8X7b	*Adineta vaga*	80579888	28897	0.036%	4028031738	1444544	0.036%
AD8X7c	*Adineta vaga*	77159840	12345	0.016%	3857128480	617118	0.016%
AD8Y24a	*Adineta vaga*	66005632	7151	0.011%	3299557790	357435	0.011%
AD8Y24b	*Adineta vaga*	72408734	8282	0.011%	3619621438	414048	0.011%
AD8Y24c	*Adineta vaga*	64429388	15062	0.023%	3220751222	753022	0.023%
AD8Y7a	*Adineta vaga*	97013708	19600	0.020%	4849556092	979854	0.020%
AD8Y7c	*Adineta vaga*	64664014	25117	0.039%	3232464688	1255458	0.039%
AD8Y7d	*Adineta vaga*	61679400	25849	0.042%	3083272030	1292098	0.042%

Supplementary Table 13. Proportion of filtered data mapping to *E. coli* OP50 23S rRNA reference.

Sample ID	Species	Total reads	Mapped reads	%	Total bases	Mapped bases	%
AD1X24b	*Adineta ricciae*	72300282	449810	0.62%	3615014100	22490500	0.62%
AD1X24c	*Adineta ricciae*	103152802	693447	0.67%	5157640100	34672350	0.67%
AD1X24d	*Adineta ricciae*	80035292	187583	0.23%	4001764600	9379150	0.23%
AD1X7a	*Adineta ricciae*	110227526	1213780	1.10%	5511376300	60689000	1.10%
AD1X7b	*Adineta ricciae*	77501642	688936	0.89%	3875082100	34446800	0.89%
AD1X7c	*Adineta ricciae*	95649472	1229311	1.29%	4782473600	61465550	1.29%
AD1Y24b	*Adineta ricciae*	80992896	1239300	1.53%	4049644800	61965000	1.53%
AD1Y24c	*Adineta ricciae*	92458758	1021638	1.11%	4622937900	51081900	1.11%
AD1Y24d	*Adineta ricciae*	73779290	603343	0.82%	3688964500	30167150	0.82%
AD1Y7a	*Adineta ricciae*	95898178	1157610	1.21%	4794908900	57880500	1.21%
AD1Y7c	*Adineta ricciae*	80801710	4634519	5.74%	4040085500	231725950	5.74%
AD1Y7d	*Adineta ricciae*	106034116	2785719	2.63%	5301705800	139285950	2.63%
AD8X24b	*Adineta vaga*	77324552	586524	0.76%	3866227600	29326200	0.76%
AD8X24c	*Adineta vaga*	97572802	1191338	1.22%	4878640100	59566900	1.22%
AD8X24d	*Adineta vaga*	81497256	870287	1.07%	4074862800	43514350	1.07%
AD8X7a	*Adineta vaga*	107985090	1423545	1.32%	5399254500	71177250	1.32%
AD8X7b	*Adineta vaga*	93504888	3410947	3.65%	4675244400	170547350	3.65%
AD8X7c	*Adineta vaga*	82552616	1022349	1.24%	4127630800	51117450	1.24%
AD8Y24a	*Adineta vaga*	70766208	685606	0.97%	3538310400	34280300	0.97%
AD8Y24b	*Adineta vaga*	76829912	721539	0.94%	3841495600	36076950	0.94%
AD8Y24c	*Adineta vaga*	70353904	1329183	1.89%	3517695200	66459150	1.89%
AD8Y7a	*Adineta vaga*	105228144	1842145	1.75%	5261407200	92107250	1.75%
AD8Y7c	*Adineta vaga*	78242264	3439601	4.40%	3912113200	171980050	4.40%
AD8Y7d	*Adineta vaga*	72992040	2979401	4.08%	3649602000	148970050	4.08%

Supplementary Table 14. Counts for significantly upregulated *A. vaga* HGT_C genes with OP50 reads mapping. Replicate count data and control/treatment means shown in columns 4–11.

Gene ID	Timepoint	Nº OP50 reads[a]	AD8X7a	AD8X7b	AD8X7c	AD8Y7a	AD8Y7c	AD8Y7d	Control mean	Treatment mean
AVAG|g19490.t1	T7	1	262	186	186	2057	1274	1164	211	1498
AVAG|g41355.t1	T7	1	156	90	92	2638	1629	1488	113	1919
AVAG|g22189.t1	T7	1	119	94	62	748	480	451	92	560
AVAG|g13392.t1	T7	1	1111	678	980	4835	3965	3101	923	3967
AVAG|g14892.t1	T7	2	56	35	38	281	154	132	43	189
AVAG|g515.t1	T7	1	136	78	83	589	504	360	99	484
AVAG|g19490.t1	T24	1	150	180	214	1869	2731	2637	182	2412
AVAG|g41355.t1	T24	1	99	118	159	3864	4746	3320	125	3977
AVAG|g2670.t1	T24	1	359	833	395	3376	4530	5152	529	4353
AVAG|g22189.t1	T24	1	84	80	78	504	811	760	80	692
AVAG|g13392.t1	T24	1	743	1184	1077	3961	4620	4580	1001	4387
AVAG|g40710.t1	T24	1	160	237	159	642	1065	1203	185	970
AVAG|g25429.t1	T24	1	152	181	210	1106	1364	1706	181	1392
AVAG|g14892.t1	T24	2	24	20	37	116	155	119	27	130

[a]Total number of filtered RNA-seq reads mapping to focal gene (i.e., that could possibly influence DE outcome).

Supplementary Table 15. Counts for significantly upregulated *A. ricciae* HGT_C genes with OP50 reads mapping. Replicate count data and control/treatment means shown in columns 4–11. The only gene for which a potential *E. coli* OP50-derived read could account for a substantial proportion of total count data (1 read mapping versus control mean of 4) is highlighted in bold and with an asterisk. This gene is most similar to a hypothetical protein with unknown function from a Delta proteobacterium and is not discussed in the text.

Gene ID	Timepoint	Nº OP50 reads[a]	AD1X7a	AD1X7b	AD1X7c	AD1Y7a	AD1Y7c	AD1Y7d	Control mean	Treatment mean
ARIC|g52925.t1	T7	1	535	419	519	7952	5499	8911	491	7454
ARIC|g13546.t1	T7	1	54	63	77	283	188	322	65	264
ARIC|g40518.t1	T7	2	1238	704	946	6698	4711	7727	963	6379
ARIC|g54144.t1	T7	1	806	380	499	6009	4404	7716	562	6043
ARIC|g21592.t1	T7	1	177	195	215	783	506	908	196	732
ARIC|g29778.t1	T7	1	302	246	282	7066	4731	7804	277	6533
ARIC|g41990.t1	T7	2	545	456	520	4622	2917	4650	507	4063
ARIC|g43360.t1	T7	1	78	142	96	3977	3416	3488	105	3627
ARIC|g46238.t1	T7	1	133	73	118	1662	1322	2282	108	1755
ARIC|g22225.t1	T7	1	675	300	406	2537	1816	3246	460	2533
ARIC|g7955.t1	T7	1	122	64	81	440	338	593	89	457
ARIC|g41469.t1	T7	1	73	47	49	720	687	1081	56	829
ARIC|g29219.t1	T7	1	268	284	314	3068	2374	3162	289	2868
ARIC|g24158.t1	T7	1	89	78	108	471	286	471	92	410
ARIC|g8513.t1	T7	1	887	831	729	4234	3877	4356	816	4156
ARIC|g31008.t1	T7	1	40	17	41	347	196	377	33	306
ARIC|g2381.t1	T7	1	585	497	640	4099	2927	4715	574	3914
ARIC|g29990.t1	T7	1	84	87	97	429	367	478	89	425
ARIC|g54564.t1	T7	1	728	466	578	4486	3526	5329	591	4447
ARIC|g41868.t1	T7	2	373	311	310	2103	1665	2808	332	2192
ARIC|g32107.t1	T7	1	193	150	203	828	538	970	182	779
ARIC|g14224.t1	T7	1	26	12	17	964	649	1046	18	887
ARIC|g50695.t1	T7	1	1146	1068	1031	7036	6718	6560	1082	6771
**ARIC|g34286.t1***	T24	1	3	6	2	39	51	52	4	47

ARIC|g29445.t1	T24	1	123	181	145	1608	1880	1821	150	1770
ARIC|g52925.t1	T24	1	343	475	465	8365	9648	7833	428	8616
ARIC|g13546.t1	T24	1	72	107	95	2229	2812	2863	91	2635
ARIC|g40518.t1	T24	2	716	789	711	6152	8634	5106	739	6631
ARIC|g54144.t1	T24	1	452	570	470	8229	10704	7402	497	8779
ARIC|g21592.t1	T24	1	82	139	198	2194	2771	3037	140	2667
ARIC|g46289.t1	T24	1	1464	2144	1446	8695	9424	8217	1685	8779
ARIC|g21234.t1	T24	1	547	750	574	2614	3663	2957	624	3078
ARIC|g29778.t1	T24	1	202	277	278	10917	12392	10689	252	11332
ARIC|g41990.t1	T24	2	407	549	495	6492	7763	7726	484	7327
ARIC|g43360.t1	T24	1	149	198	107	8818	16137	11968	151	12308
ARIC|g46238.t1	T24	1	82	138	165	23924	26912	24607	128	25148
ARIC|g22225.t1	T24	1	393	576	395	2020	2556	1629	455	2068
ARIC|g7955.t1	T24	1	30	54	65	2593	3163	2239	50	2665
ARIC|g41469.t1	T24	1	39	59	33	715	784	521	44	673
ARIC|g29219.t1	T24	1	169	213	108	2305	2854	2591	163	2583
ARIC|g24158.t1	T24	1	55	68	81	764	983	986	68	911
ARIC|g8513.t1	T24	1	1142	1739	1526	8731	13435	10373	1469	10846
ARIC|g31008.t1	T24	1	35	49	17	462	649	591	34	567
ARIC|g2381.t1	T24	1	466	560	522	5141	6232	5287	516	5553
ARIC|g9992.t1	T24	1	118	174	110	488	640	578	134	569
ARIC|g29990.t1	T24	1	80	136	110	2902	3499	3497	109	3299
ARIC|g54564.t1	T24	1	444	544	436	1813	2220	1572	475	1868
ARIC|g18096.t1	T24	1	140	214	163	860	922	734	172	839
ARIC|g41868.t1	T24	2	240	460	377	1534	2021	1408	359	1654
ARIC|g32107.t1	T24	1	115	168	149	3206	3534	3706	144	3482
ARIC|g14224.t1	T24	1	5	22	15	980	1002	905	14	962
ARIC|g31203.t1	T24	1	247	416	307	1096	1420	900	323	1139
ARIC|g50695.t1	T24	1	1469	2280	1902	11853	18834	13904	1884	14864

[a] Total number of filtered RNA-seq reads mapping to focal gene (i.e., that could possibly influence DE outcome).

Supplementary References

1. Segers, H. & Shiel, R. Tale of a sleeping beauty: a new and easily cultured model organism for experimental studies on bdelloid rotifers. *Rotifera X* 141–145 (2005).
2. Davis, H. A new callidina: With the result of experiments on the desiccation of rotifers. *Mon. Microsc. J.* **9**, 201–209 (1873).
3. Örstan, A. The trouble with *Adineta vaga* (Davis, 1873): a common rotifer that cannot be identified (Rotifera: Bdelloidea: Adinetidae). *Zootaxa* **4830**, 597–600 (2020).
4. Ricci, C. Culturing of some bdelloid rotifers. *Hydrobiologia* **112**, 45–51 (1984).
5. Mark Welch, D. B. & Meselson, M. Oocyte nuclear DNA content and GC proportion in rotifers of the anciently asexual Class Bdelloidea. *Biol. J. Linn. Soc. Lond.* **79**, 85–91 (2003).
6. Pouchkina-Stantcheva, N. N. *et al.* Functional divergence of former alleles in an ancient asexual invertebrate. *Science* **318**, 268–271 (2007).
7. Flot, J.-F. *et al.* Genomic evidence for ameiotic evolution in the bdelloid rotifer *Adineta vaga*. *Nature* **500**, 453–457 (2013).
8. Wilson, C. G., Nowell, R. W. & Barraclough, T. G. Cross-contamination explains "Inter and intraspecific horizontal genetic transfers" between asexual bdelloid rotifers. *Curr. Biol.* **28**, 2436–2444 (2018).
9. Nowell, R. W. *et al.* Comparative genomics of bdelloid rotifers: Insights from desiccating and nondesiccating species. *PLoS Biol.* **16**, e2004830 (2018).
10. Barron, G. L. A new genus, *Rotiferophthora*, to accommodate the *Diheterospora*-like endoparasites of rotifers. *Can. J. Bot.* **69**, 494–502 (1991).
11. Wilson, C. G. Desiccation-tolerance in bdelloid rotifers facilitates spatiotemporal escape from multiple species of parasitic fungi. *Biol. J. Linn. Soc. Lond.* **104**, 564–574 (2011).
12. Barron, G. L. Fungal parasites of bdelloid rotifers: *Diheterospora*. *Can. J. Bot.* **63**, 211–222 (1985).
13. Jenkins, D. G. & Underwood, M. O. Zooplankton may not disperse readily in wind, rain, or waterfowl. in *Rotifera VIII: A Comparative Approach* 15–21 (Springer Netherlands, 1998). doi:10.1007/978-94-011-4782-8_3.
14. Fontaneto, D., Barraclough, T. G., Chen, K., Ricci, C. & Herniou, E. A. Molecular evidence for broad-scale distributions in bdelloid rotifers: everything is not everywhere but most things are very widespread. *Mol. Ecol.* **17**, 3136–3146 (2008).
15. Fontaneto, D. *et al.* Cryptic diversity in the genus Adineta Hudson & Gosse, 1886 (Rotifera: Bdelloidea: Adinetidae): a DNA taxonomy approach. *Hydrobiologia* **662**, 27–33 (2011).
16. Wilson, C. G. & Sherman, P. W. Spatial and temporal escape from fungal parasitism in natural communities of anciently asexual bdelloid rotifers. *Proc. Biol. Sci.* **280**, 20131255 (2013).
17. McCallum, H. *et al.* Breaking beta: deconstructing the parasite transmission function. *Philos. Trans. R. Soc. Lond. B Biol. Sci.* **372**, (2017).
18. Wilson, C. G. & Sherman, P. W. Anciently asexual bdelloid rotifers escape lethal fungal parasites by drying up and blowing away. *Science* **327**, 574–576 (2010).
19. Ricci, C. & Caprioli, M. Anhydrobiosis in bdelloid species, populations and individuals. *Integr. Comp. Biol.* **45**, 759–763 (2005).
20. Quast, C. *et al.* The SILVA ribosomal RNA gene database project: improved data processing and web-based tools. *Nucleic Acids Res.* **41**, D590-6 (2013).
21. Dobin, A. *et al.* STAR: ultrafast universal RNA-seq aligner. *Bioinformatics* **29**, 15–21 (2013).
22. Andrews, S. *FastQC: A quality-control tool for high-throughput sequence data*. (2015).
23. Ewels, P., Magnusson, M., Lundin, S. & Käller, M. MultiQC: summarize analysis results for multiple tools and samples in a single report. *Bioinformatics* **32**, 3047–3048 (2016).
24. Mark Welch, D. B., Mark Welch, J. L. & Meselson, M. Evidence for degenerate tetraploidy in bdelloid rotifers. *Proc. Natl. Acad. Sci. U. S. A.* **105**, 5145–5149 (2008).
25. Hur, J. H., Van Doninck, K., Mandigo, M. L. & Meselson, M. Degenerate tetraploidy was established before bdelloid rotifer families diverged. *Mol. Biol. Evol.* **26**, 375–383 (2009).
26. Grabherr, M. G. *et al.* Full-length transcriptome assembly from RNA-Seq data without a reference genome. *Nat. Biotechnol.* **29**, 644–652 (2011).

27. Haas, B. J. *et al.* De novo transcript sequence reconstruction from RNA-seq using the Trinity platform for reference generation and analysis. *Nat. Protoc.* **8**, 1494–1512 (2013).
28. Patro, R., Duggal, G., Love, M. I., Irizarry, R. A. & Kingsford, C. Salmon provides fast and bias-aware quantification of transcript expression. *Nat. Methods* **14**, 417–419 (2017).
29. Love, M. I., Huber, W. & Anders, S. Moderated estimation of fold change and dispersion for RNA-seq data with DESeq2. *Genome Biol.* **15**, 550 (2014).
30. Benjamini, Y. & Hochberg, Y. Controlling the False Discovery Rate: A practical and powerful approach to multiple testing. *J. R. Stat. Soc. Series B Stat. Methodol.* **57**, 289–300 (1995).
31. Yoon, S. & Nam, D. Gene dispersion is the key determinant of the read count bias in differential expression analysis of RNA-seq data. *BMC Genomics* **18**, 408 (2017).
32. Soneson, C. & Robinson, M. D. Bias, robustness and scalability in single-cell differential expression analysis. *Nat. Methods* **15**, 255–261 (2018).
33. Robinson, M. D., McCarthy, D. J. & Smyth, G. K. edgeR: a Bioconductor package for differential expression analysis of digital gene expression data. *Bioinformatics* **26**, 139–140 (2010).
34. McCarthy, D. J., Chen, Y. & Smyth, G. K. Differential expression analysis of multifactor RNA-Seq experiments with respect to biological variation. *Nucleic Acids Res.* **40**, 4288–4297 (2012).
35. Chen, Y., Lun, A. T. L. & Smyth, G. K. From reads to genes to pathways: differential expression analysis of RNA-Seq experiments using Rsubread and the edgeR quasi-likelihood pipeline. *F1000Res.* **5**, 1438 (2016).
36. Ritchie, M. E. *et al.* limma powers differential expression analyses for RNA-sequencing and microarray studies. *Nucleic Acids Res.* **43**, e47 (2015).
37. Li, H. Minimap2: pairwise alignment for nucleotide sequences. *Bioinformatics* **34**, 3094–3100 (2018).
38. Simão, F. A., Waterhouse, R. M., Ioannidis, P., Kriventseva, E. V. & Zdobnov, E. M. BUSCO: assessing genome assembly and annotation completeness with single-copy orthologs. *Bioinformatics* **31**, 3210–3212 (2015).
39. Emms, D. M. & Kelly, S. OrthoFinder: phylogenetic orthology inference for comparative genomics. *Genome Biol.* **20**, 238 (2019).
40. Han, J. *et al.* The genome of the marine monogonont rotifer *Brachionus plicatilis*: Genome-wide expression profiles of 28 cytochrome P450 genes in response to chlorpyrifos and 2-ethyl-phenanthrene. *Aquat. Toxicol.* **214**, 105230 (2019).
41. Nowell, R. W. *et al.* Evolutionary dynamics of transposable elements in bdelloid rotifers. *Elife* **10**, e63194 (2021).
42. Jaron, K. S. *et al.* Genomic features of parthenogenetic animals. *J. Hered.* **112**, 19–33 (2021).
43. UniProt Consortium. UniProt: a worldwide hub of protein knowledge. *Nucleic Acids Res.* **47**, D506–D515 (2019).
44. Buchfink, B., Xie, C. & Huson, D. H. Fast and sensitive protein alignment using DIAMOND. *Nat. Methods* **12**, 59–60 (2015).
45. Boschetti, C. *et al.* Biochemical diversification through foreign gene expression in bdelloid rotifers. *PLoS Genet.* **8**, e1003035 (2012).
46. Koutsovoulos, G. *et al.* No evidence for extensive horizontal gene transfer in the genome of the tardigrade Hypsibius dujardini. *Proceedings of the National Academy of Sciences of the United States of America* vol. 113 5053–5058 (2016).
47. Simion, P. *et al.* Chromosome-level genome assembly reveals homologous chromosomes and recombination in asexual rotifer *Adineta vaga*. *Sci Adv* **7**, eabg4216 (2021).
48. Nguyen, L.-T., Schmidt, H. A., von Haeseler, A. & Minh, B. Q. IQ-TREE: a fast and effective stochastic algorithm for estimating maximum-likelihood phylogenies. *Mol. Biol. Evol.* **32**, 268–274 (2015).
49. Kalyaanamoorthy, S., Minh, B. Q., Wong, T. K. F., von Haeseler, A. & Jermiin, L. S. ModelFinder: fast model selection for accurate phylogenetic estimates. *Nat. Methods* **14**, 587–589 (2017).
50. Minh, B. Q. *et al.* IQ-TREE 2: New models and efficient methods for phylogenetic inference in the genomic era. *Mol. Biol. Evol.* (2020) doi:10.1093/molbev/msaa015.

51. El-Gebali, S. *et al.* The Pfam protein families database in 2019. *Nucleic Acids Res.* **47**, D427–D432 (2019).
52. Altschul, S. F., Gish, W., Miller, W., Myers, E. W. & Lipman, D. J. Basic local alignment search tool. *J. Mol. Biol.* **215**, 403–410 (1990).
53. Petersen, T. N., Brunak, S., von Heijne, G. & Nielsen, H. SignalP 4.0: discriminating signal peptides from transmembrane regions. *Nat. Methods* **8**, 785–786 (2011).
54. Krogh, A., Larsson, B., von Heijne, G. & Sonnhammer, E. L. Predicting transmembrane protein topology with a hidden Markov model: application to complete genomes. *J. Mol. Biol.* **305**, 567–580 (2001).
55. Jones, P. *et al.* InterProScan 5: genome-scale protein function classification. *Bioinformatics* **30**, 1236–1240 (2014).
56. Bryant, D. M. *et al.* A tissue-mapped axolotl de novo transcriptome enables identification of limb regeneration factors. *Cell Rep.* **18**, 762–776 (2017).
57. Young, M. D., Wakefield, M. J., Smyth, G. K. & Oshlack, A. Gene ontology analysis for RNA-seq: accounting for selection bias. *Genome Biol.* **11**, R14 (2010).
58. Supek, F., Bošnjak, M., Škunca, N. & Šmuc, T. REVIGO summarizes and visualizes long lists of gene ontology terms. *PLoS One* **6**, e21800 (2011).
59. Hecox-Lea, B. J. & Mark Welch, D. B. Evolutionary diversity and novelty of DNA repair genes in asexual bdelloid rotifers. *BMC Evol. Biol.* **18**, 177 (2018).
60. Moris, V. C. *et al.* Ionizing radiation responses appear incidental to desiccation responses in the bdelloid rotifer *Adineta vaga*. *BMC Biol.* **22**, 11 (2024).
61. Blin, K. *et al.* antiSMASH 6.0: improving cluster detection and comparison capabilities. *Nucleic Acids Res.* **49**, W29–W35 (2021).
62. Röttig, M. *et al.* NRPSpredictor2--a web server for predicting NRPS adenylation domain specificity. *Nucleic Acids Res.* **39**, W362-7 (2011).
63. Gladyshev, E. A. & Arkhipova, I. R. Telomere-associated endonuclease-deficient *Penelope*-like retroelements in diverse eukaryotes. *Proc. Natl. Acad. Sci. U. S. A.* **104**, 9352–9357 (2007).
64. Buchfink, B., Reuter, K. & Drost, H.-G. Sensitive protein alignments at tree-of-life scale using DIAMOND. *Nat. Methods* **18**, 366–368 (2021).
65. Suring, W. *et al.* Nonribosomal Peptide Synthetases in Animals. *Genes* **14**, 1741 (2023).
66. Hoang, D. T., Chernomor, O., von Haeseler, A., Minh, B. Q. & Vinh, L. S. UFBoot2: Improving the Ultrafast Bootstrap Approximation. *Mol. Biol. Evol.* **35**, 518–522 (2018).
67. Guindon, S. *et al.* New algorithms and methods to estimate maximum-likelihood phylogenies: assessing the performance of PhyML 3.0. *Syst. Biol.* **59**, 307–321 (2010).
68. Revell, L. J. phytools: an R package for phylogenetic comparative biology (and other things). *Methods Ecol. Evol.* **3**, 217–223 (2012).
69. Paradis, E. & Schliep, K. ape 5.0: an environment for modern phylogenetics and evolutionary analyses in R. *Bioinformatics* **35**, 526–528 (2019).
70. Zierep, P. F., Ceci, A. T., Dobrusin, I., Rockwell-Kollmann, S. C. & Günther, S. SeMPI 2.0-A web server for PKS and NRPS predictions combined with metabolite screening in natural product databases. *Metabolites* **11**, (2020).
71. Du, D. et al. Multidrug efflux pumps: structure, function and regulation. *Nat. Rev. Microbiol.* **16**, 523–539 (2018).
72. Bernhardt, R. Cytochromes P450 as versatile biocatalysts. J. Biotechnol. **124**, 128–145 (2006).
