# Supplementary material for: Bdelloid rotifers deploy horizontally acquired biosynthetic genes against a fungal pathogen
Source: Nat Commun. 2024 Jul 18;15:5787. doi: 10.1038/s41467-024-49919-1 (PMC11258130; doi:10.1038/s41467-024-49919-1)
Supplement: Supplementary file 3 — Reporting Summary [file 41467_2024_49919_MOESM3_ESM.pdf]

nature portfolio

Corresponding author(s): Christopher G. Wilson

Last updated by author(s): 28/05/2024

Reporting Summary

Nature Portfolio wishes to improve the reproducibility of the work that we publish. This form provides structure for consistency and transparency in reporting. For further information on Nature Portfolio policies, see our Editorial Policies and the Editorial Policy Checklist.

Statistics

For all statistical analyses, confirm that the following items are present in the figure legend, table legend, main text, or Methods section.

n/a	Confirmed	
☐	☒	The exact sample size (*n*) for each experimental group/condition, given as a discrete number and unit of measurement
☒	☐	A statement on whether measurements were taken from distinct samples or whether the same sample was measured repeatedly
☐	☒	The statistical test(s) used AND whether they are one- or two-sided<br>*Only common tests should be described solely by name; describe more complex techniques in the Methods section.*
☐	☒	A description of all covariates tested
☐	☒	A description of any assumptions or corrections, such as tests of normality and adjustment for multiple comparisons
☐	☒	A full description of the statistical parameters including central tendency (e.g. means) or other basic estimates (e.g. regression coefficient) AND variation (e.g. standard deviation) or associated estimates of uncertainty (e.g. confidence intervals)
☐	☒	For null hypothesis testing, the test statistic (e.g. *F*, *t*, *r*) with confidence intervals, effect sizes, degrees of freedom and *P* value noted<br>*Give P values as exact values whenever suitable.*
☒	☐	For Bayesian analysis, information on the choice of priors and Markov chain Monte Carlo settings
☒	☐	For hierarchical and complex designs, identification of the appropriate level for tests and full reporting of outcomes
☐	☒	Estimates of effect sizes (e.g. Cohen's *d*, Pearson's *r*), indicating how they were calculated

Our web collection on statistics for biologists contains articles on many of the points above.

Software and code

Policy information about availability of computer code

Data collection	Manufacturer's software provided by Illumina was used for sequence data collection
Data analysis	BBTools 'bbduk' v38.73 for read quality and adapter trimming;<br>BBTools 'tadpole' v38.73 for read error correction;<br>BBTools 'bbmap' v38.73 for RNA-seq read mapping;<br>STAR v2.7.3a for RNA-seq read mapping;<br>FastQC v0.11.6 and MultiQC v1.9 for sequence data QC and visualisation;<br>Trinity v2.8.9 for transcriptome assembly;<br>BUSCO v5 for gene completeness scoring;<br>Salmon 'quant' v0.14.1 for transcriptome quantification;<br>collate_DE_results.pl (custom script, https://github.com/reubwn/bdelloid-immunity) for collating HGT and DE results;<br>bootstrap_collate_DE_results.pl (custom script, https://github.com/reubwn/bdelloid-immunity) for generating bootstrap samples of DE results;<br>get_expression_of_orthologs.pl (custom script, https://github.com/reubwn/bdelloid-immunity) for obtaining expression values for identified pairs of orthologs;<br>R version 3.6.1 for core statistical analyses;<br>DESeq2_1.26.0, edgeR_3.28.1 and limma_3.42.2 for differential expression analysis;<br>Minimap2 v2.17 for transcriptome mapping;<br>Trinotate v3.2.0, BLAST v2.10.1+, HMMER v3.3, SignalP v4.1 and TMHMM v2.0 for functional annotation of protein sequences;<br>goseq_1.38.0 for GO analyses;

Diamond 'blastp' v0.9.21 for sequence alignment;
IQTREE v1.6.12 for phylogenetic reconstruction;
OrthoFinder v2.3.12 for orthology reconstruction;
AntiSMASH v6.1.1 for NRP/PKS annotation;
BEDTools v2.29.2 for genome context analyses;
SEMPI v2.0 and ChemDraw JS (version 19.0.0-CDJS-19.0.x.9+da9bec968) for secondary metabolite prediction and visualisation.

For manuscripts utilizing custom algorithms or software that are central to the research but not yet described in published literature, software must be made available to editors and reviewers. We strongly encourage code deposition in a community repository (e.g. GitHub). See the Nature Portfolio guidelines for submitting code & software for further information.

Data

Policy information about availability of data

All manuscripts must include a data availability statement. This statement should provide the following information, where applicable:
- Accession codes, unique identifiers, or web links for publicly available datasets
- A description of any restrictions on data availability
- For clinical datasets or third party data, please ensure that the statement adheres to our policy

All raw sequencing data generated by this study have been deposited in the relevant International Nucleotide Sequence Database Collaboration (INSDC) database with the BioProject ID PRJEB39927, with the SRA run accessions ERR4469891, ERR4469902–8, ERR4471099–102, ERR4471104–11, ERR4471113–6 (see Supplementary Table 8). This study also analysed publicly available data from BioProjects PRJEB1171, PRJEB23547, and PRJEB43248 (genome assemblies, gene annotations, TE annotations), and PRJNA494578 (RNA-seq data for the desiccation test).

Research involving human participants, their data, or biological material

Policy information about studies with human participants or human data. See also policy information about sex, gender (identity/presentation), and sexual orientation and race, ethnicity and racism.

Reporting on sex and gender	NA
Reporting on race, ethnicity, or other socially relevant groupings	NA
Population characteristics	NA
Recruitment	NA
Ethics oversight	NA

Note that full information on the approval of the study protocol must also be provided in the manuscript.

Field-specific reporting

Please select the one below that is the best fit for your research. If you are not sure, read the appropriate sections before making your selection.

☒ Life sciences ☐ Behavioural & social sciences ☐ Ecological, evolutionary & environmental sciences

For a reference copy of the document with all sections, see nature.com/documents/nr-reporting-summary-flat.pdf

Life sciences study design

All studies must disclose on these points even when the disclosure is negative.

Sample size	Rotifer populations were subdivided to yield 16 replicates for each species, with approximately 1000 animals per sample for A. ricciae and 600 for A. vaga. Tubes were then randomly allocated to receive either live or irradiated pathogen spores of the fungal pathogen R. globospora, and to have RNA extracted either 7 or 24 hours later, with each combination replicated four times. Three of the four replicates in each category were taken forward to RNA-seq because this is the minimum standard for differential expression transcriptomics.
Data exclusions	Unwanted ribosomal RNA (rRNA) reads were removed by mapping to the SILVA rRNA database using BBTools 'bbmap'. Contaminant reads derived from either the bacterial rotifer food (E. coli strain OP50) or from the fungal pathogen itself were removed using a similar approach, mapping to sequenced genomes of fungi in the family Clavicipitaceae (NCBI taxid 34397) or to the OP50 genome (see Supplementary Methods for further details). These are standard exclusions for transcriptomic analyses tp ensure only target reads are included, and were pre-established..Selection of the three RNA replicates to take forward to sequencing was on the basis of initial RNA quality metrics (highest yield and purity).
Replication	Core statistical analyses were replicated using de novo transcriptomes assembled and annotated directly from RNA-seq data, rather than based on previously published genome data. The same conclusions are reached in both cases (see Supplementary Methods for further details). Transcriptomic analyses were replicated using three different RNA-seq analysis pipelines and with multiple significance thresholds

	with nearly identical results (detailed in Supplementary Methods).
Randomization	Tubes were randomly allocated to receive either live or irradiated pathogen spores.
Blinding	The investigator who conducted the statistical analyses (Reuben W. Nowell) was blind to the group allocation (pathogen vs control) of RNA-seq libraries until after the primary analyses were conducted.

Behavioural & social sciences study design

All studies must disclose on these points even when the disclosure is negative.

Study description	NA
Research sample	NA
Sampling strategy	NA
Data collection	NA
Timing	NA
Data exclusions	NA
Non-participation	NA
Randomization	NA

Ecological, evolutionary & environmental sciences study design

All studies must disclose on these points even when the disclosure is negative.

Study description	NA
Research sample	NA
Sampling strategy	NA
Data collection	NA
Timing and spatial scale	NA
Data exclusions	NA
Reproducibility	NA
Randomization	NA
Blinding	NA

Did the study involve field work? ☐ Yes ☐ No

Field work, collection and transport

Field conditions	NA
Location	NA
Access & import/export	NA
Disturbance	NA

Reporting for specific materials, systems and methods

We require information from authors about some types of materials, experimental systems and methods used in many studies. Here, indicate whether each material, system or method listed is relevant to your study. If you are not sure if a list item applies to your research, read the appropriate section before selecting a response.

Materials & experimental systems

n/a	Involved in the study
☒	☐ Antibodies
☒	☐ Eukaryotic cell lines
☒	☐ Palaeontology and archaeology
☐	☒ Animals and other organisms
☒	☐ Clinical data
☒	☐ Dual use research of concern
☒	☐ Plants

Methods

n/a	Involved in the study
☒	☐ ChIP-seq
☒	☐ Flow cytometry
☒	☐ MRI-based neuroimaging

Antibodies

Antibodies used	NA
Validation	NA

Eukaryotic cell lines

Policy information about cell lines and Sex and Gender in Research

Cell line source(s)	NA
Authentication	NA
Mycoplasma contamination	NA
Commonly misidentified lines (See ICLAC register)	NA

Palaeontology and Archaeology

Specimen provenance	NA
Specimen deposition	NA
Dating methods	NA

☐ Tick this box to confirm that the raw and calibrated dates are available in the paper or in Supplementary Information.

Ethics oversight	NA

Note that full information on the approval of the study protocol must also be provided in the manuscript.

Animals and other research organisms

Policy information about studies involving animals; ARRIVE guidelines recommended for reporting animal research, and Sex and Gender in Research

Laboratory animals	Freshwater invertebrates (Rotifera; Bdelloidea): Adineta vaga strain AD008 and Adineta ricciae strain AD001.
Wild animals	The study did not involve wild animals.
Reporting on sex	Sex-based analyses were not performed because bdelloid rotifers are asexuals and are therefore entirely female (no males).
Field-collected samples	The study did not involve samples collected from the field.

Ethics oversight | No ethical approval or guidance is required for microscopic invertebrate animals.

Note that full information on the approval of the study protocol must also be provided in the manuscript.

Clinical data

Policy information about clinical studies

All manuscripts should comply with the ICMJE guidelines for publication of clinical research and a completed CONSORT checklist must be included with all submissions.

Clinical trial registration	NA
Study protocol	NA
Data collection	NA
Outcomes	NA

Dual use research of concern

Policy information about dual use research of concern

Hazards

Could the accidental, deliberate or reckless misuse of agents or technologies generated in the work, or the application of information presented in the manuscript, pose a threat to:

No	Yes	
☐	☐	Public health
☐	☐	National security
☐	☐	Crops and/or livestock
☐	☐	Ecosystems
☐	☐	Any other significant area

Experiments of concern

Does the work involve any of these experiments of concern:

No	Yes	
☐	☐	Demonstrate how to render a vaccine ineffective
☐	☐	Confer resistance to therapeutically useful antibiotics or antiviral agents
☐	☐	Enhance the virulence of a pathogen or render a nonpathogen virulent
☐	☐	Increase transmissibility of a pathogen
☐	☐	Alter the host range of a pathogen
☐	☐	Enable evasion of diagnostic/detection modalities
☐	☐	Enable the weaponization of a biological agent or toxin
☐	☐	Any other potentially harmful combination of experiments and agents

Plants

Seed stocks	NA
Novel plant genotypes	NA
Authentication	NA

ChIP-seq

Data deposition

☐ Confirm that both raw and final processed data have been deposited in a public database such as GEO.

☐ Confirm that you have deposited or provided access to graph files (e.g. BED files) for the called peaks.

Data access links<br>*May remain private before publication.*	NA
Files in database submission	NA
Genome browser session<br>(e.g. UCSC)	NA

Methodology

Replicates	NA
Sequencing depth	NA
Antibodies	NA
Peak calling parameters	NA
Data quality	NA
Software	NA

Flow Cytometry

Plots

Confirm that:

- [ ] The axis labels state the marker and fluorochrome used (e.g. CD4-FITC).
- [ ] The axis scales are clearly visible. Include numbers along axes only for bottom left plot of group (a 'group' is an analysis of identical markers).
- [ ] All plots are contour plots with outliers or pseudocolor plots.
- [ ] A numerical value for number of cells or percentage (with statistics) is provided.

Methodology

Sample preparation	NA
Instrument	NA
Software	NA
Cell population abundance	NA
Gating strategy	NA

- [ ] Tick this box to confirm that a figure exemplifying the gating strategy is provided in the Supplementary Information.

Magnetic resonance imaging

Experimental design

Design type	NA
Design specifications	NA
Behavioral performance measures	NA

Acquisition

Imaging type(s)	NA
Field strength	NA
Sequence & imaging parameters	NA
Area of acquisition	NA

Diffusion MRI ☐ Used ☐ Not used

Preprocessing

Preprocessing software	NA
Normalization	NA
Normalization template	NA
Noise and artifact removal	NA
Volume censoring	NA

Statistical modeling & inference

Model type and settings	NA
Effect(s) tested	NA

Specify type of analysis: ☐ Whole brain ☐ ROI-based ☐ Both

Statistic type for inference (See Eklund et al. 2016)	NA
Correction	NA

Models & analysis

n/a	Involved in the study
☐	☐ Functional and/or effective connectivity
☐	☐ Graph analysis
☐	☐ Multivariate modeling or predictive analysis

Functional and/or effective connectivity	NA
Graph analysis	NA
Multivariate modeling and predictive analysis	NA
