# Supplementary material for: Bdelloid rotifers deploy horizontally acquired biosynthetic genes against a fungal pathogen
Source: Nat Commun. 2024 Jul 18;15:5787. doi: 10.1038/s41467-024-49919-1 (PMC11258130; doi:10.1038/s41467-024-49919-1)
Supplement: Supplementary file 4 — Description of Additional Supplementary Files [file 41467_2024_49919_MOESM4_ESM.pdf]

Description of Additional Supplementary Files

File Name: Supplementary Data 1

Description: Gene expression data: raw counts, TMM normalized expression, and collated results. (.xlsx)

File Name: Supplementary Data 2

Description: Mean CPM per orthologous group. (.xlsx)

File Name: Supplementary Data 3

Description: GO enrichment results for HGTc biotic stress. (.xlsx)

File Name: Supplementary Data 4

Description: GO enrichment results for de novo transcriptomes with no HGTc filter. (.xlsx)

File Name: Supplementary Data 5

Description: GO enrichment results for HGTc abiotic stress. (.xlsx)

File Name: Supplementary Data 6

Description: GOseq output for upregulated genes. (.xlsx)

File Name: Supplementary Data 7

Description: NRP/PKS inventories by 3-domain HMM and GO annotation sets. (.xlsx)

File Name: Supplementary Data 8

Description: Putative NRP/PKS matches to Uniref90 output. (.xlsx)

File Name: Supplementary Data 9

Description: Manual re-annotation and cross-check of putative biosynthetic clusters in Av13 and Av20 assemblies. (.xlsx)

File Name: Supplementary Data 10

Description: Highlighted genes inventory. (.xlsx)

File Name: Supplementary Data 11

Description: SeMPI 2.0 metabolite prediction project outputs. (.zip)

File Name: Supplementary Data 12

Description: Upregulated HGTc copy number and orthology. (.xlsx)
