# Supplementary material for: Bdelloid rotifers deploy horizontally acquired biosynthetic genes against a fungal pathogen
Source: Nat Commun. 2024 Jul 18;15:5787. doi: 10.1038/s41467-024-49919-1 (PMC11258130; doi:10.1038/s41467-024-49919-1)
Supplement: Supplementary file 15 — Supplementary Data 11 [file 41467_2024_49919_MOESM15_ESM.zip › project AVAG00146 scaffold/genome_browser/cluster_1.0/index.html]

genome\_browser\_dummy
